# Modeling X-Linked Ancestral Origins in Multiparental Populations

**DOI:** 10.1534/g3.114.016154

**Published:** 2015-03-04

**Authors:** Chaozhi Zheng

**Affiliations:** Biometris, Wageningen University and Research Centre, The Netherlands

**Keywords:** advanced intercross lines (AIL), Collaborative Cross (CC), Diversity Outcross (DO), *Drosophila* synthetic population resource (DSPR), map expansion, MPP, multiparental populations, Multiparent Advanced Generation Inter-Cross (MAGIC)

## Abstract

The models for the mosaic structure of an individual’s genome from multiparental populations have been developed primarily for autosomes, whereas X chromosomes receive very little attention. In this paper, we extend our previous approach to model ancestral origin processes along two X chromosomes in a mapping population, which is necessary for developing hidden Markov models in the reconstruction of ancestry blocks for X-linked quantitative trait locus mapping. The model accounts for the joint recombination pattern, the asymmetry between maternally and paternally derived X chromosomes, and the finiteness of population size. The model can be applied to various mapping populations such as the advanced intercross lines (AIL), the Collaborative Cross (CC), the heterogeneous stock (HS), the Diversity Outcross (DO), and the *Drosophila* synthetic population resource (DSPR). We further derive the map expansion, density (per Morgan) of recombination breakpoints, in advanced intercross populations with *L* inbred founders under the limit of an infinitely large population size. The analytic results show that for X chromosomes the genetic map expands linearly at a rate (per generation) of two-thirds times 1 – 10/(9*L*) for the AIL, and at a rate of two-thirds times 1 – 1/*L* for the DO and the HS, whereas for autosomes the map expands at a rate of 1 – 1/*L* for the AIL, the DO, and the HS.

There have been recently designed quantitative trait locus (QTL) mapping populations with either multiple parents to increase the genetic diversity of the founder population, or many intercross generations to improve the mapping resolution by accumulating historical recombination events. Some examples include the Collaborative Cross (CC) (Churchill *et al.* 2004), the advanced intercross lines (AIL) (Darvasi and Soller 1995), the heterogeneous stock (HS) (Mott *et al.* 2000), the diversity outcross (DO) (Svenson *et al.* 2012), and the *Drosophila* synthetic population resource (DSPR) (King *et al.* 2012). The CC can be regarded as a set of eight-way recombinant inbred lines (RIL) by sibling mating, where eight founders of each line are permuted.

The genomes of individuals in QTL mapping populations are random mosaics of the founders’ genomes. The QTL mapping generally necessitates the reconstruction of these genome blocks along two homologous chromosomes of a sampled individual from available genotype data. Such reconstruction is often performed under a hidden Markov model (HMM) with the latent state being the pair of ancestral origins at a locus, where the transition probability of ancestral origins between two loci, or the two-locus diplotype (two-haplotype) probabilities are required.

Modeling ancestral origins along a pair of autosomal chromosomes has been well developed recently. Broman (2012a) extended the approach of Haldane and Waddington (1931) from the two-way to four- and eight-way RIL by sibling mating and provided recipes for calculating autosomal two-locus diplotype probabilities numerically. Johannes and Colome-Tatche (2011) derived autosomal two-locus diplotype probabilities for the two-way RIL by selfing. Zheng *et al.* (2014) described a general modeling framework for ancestral origins that can be applied to autosomes in various mapping populations such as the RIL by selfing or sibling mating and the AIL.

A special treatment is required for modeling ancestral origins along a pair of X chromosomes. Haldane and Waddington (1931) derived the recurrence relations of the X-linked two-locus diplotype probabilities for the two-way RIL by sibling mating and the bi-parental repeated parent-offspring mating, and their closed form solutions for the final homozygous lines. Broman (2005) extended the solutions to the two- and three-locus haplotype probabilities for the two, four, or eight-way RIL by sibling mating. Broman (2012b) derived the X-linked two-locus haplotype probabilities in advanced intercross populations including the AIL, the HS, and the DO, assuming an infinitely large population size.

In this paper, we extend our previous work (Zheng *et al.* 2014) to model the ancestral origins along a pair of X chromosomes in a finite mapping population. This extension also builds on the theory of junctions in inbreeding (Fisher 1949, 1954). A junction is defined as a boundary point of genome blocks on chromosomes where two distinct ancestral origins meet, and the boundary points that occur at the same location along multiple chromosomes are counted as a single junction. The map expansion is the expected junction density (per Morgan) on a maternally or paternally derived X chromosome, denoted by $R^{m}$ or $R^{p}$, respectively. We denote by $\rho^{mp}$ the overall junction density along the XX chromosomes of a female, and it can be used as a measure of X-linked QTL mapping resolution (Darvasi and Soller 1995; Weller and Soller 2004).

The key feature of this extension is to account for the asymmetry between maternally and paternally derived X chromosomes because the latter did not experience any crossover events with Y chromosomes. We first present a model framework for X-linked ancestral origins, where the recurrent relations are derived for various junction densities including the map expansions $R^{m}$ and $R^{p}$. Then, we derive the closed form solutions for these expected densities in mapping populations including the RIL by sibling mating, the AIL, the HS, the DO, and the DSPR; they are evaluated by forward simulation studies. Lastly, we discuss the model assumptions and the implications of the analytic results on haplotype reconstructions and breeding designs.

## A model for X-linked ancestral origins

### Assumptions and notation

Consider a dioecious population with two separate sexes: homogametic females with sex chromosomes XX and heterogametic males with sex chromosomes XY. There are no recombination events between X and Y, and thus we ignore the pseudoautosomal regions on the XY chromosomes. As in most mammals and some insects (*Drosophila*), some flowering species, such as white campion (*Silene latifolia*), papaya (*Carica papaya*), and asparagus (*Asparagus officianalis*), have the XY sex determination system (Ming and Moore 2007). The dioecious population was founded in generation 0, and it has nonoverlapping generations. There are no natural or artificial selections since the founder population. The mating schemes of producing the next generation are random, and they may vary from one generation to the next. The assignments of offspring genders are assumed to be independent of mating schemes.

**Figure 1:**
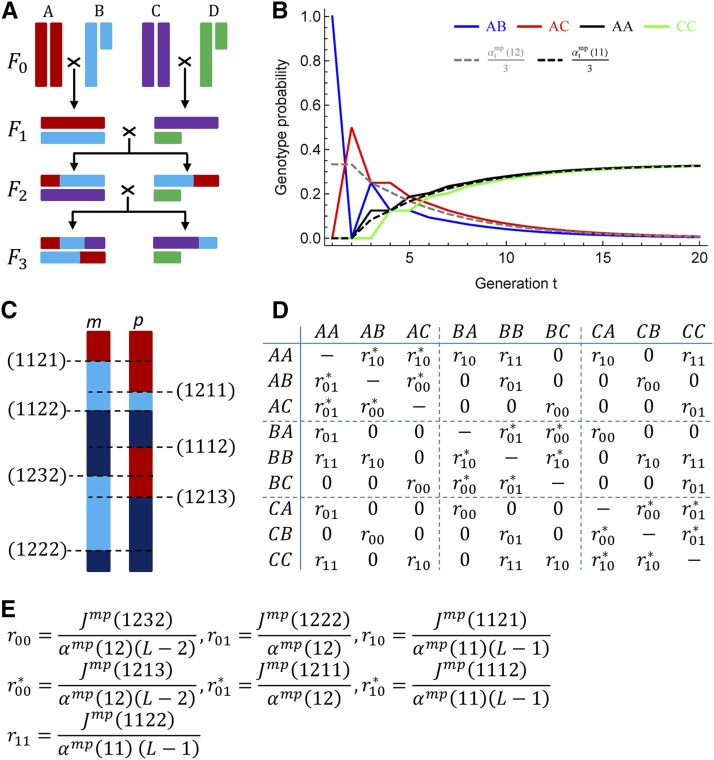
The continuous time Markov chain (CTMC) of X-linked ancestry blocks in the four-way recombinant inbred lines (RILs) by sibling mating. (A) One realization of ancestry blocks in the four-way RIL with generation up to *F*_3_. The sex chromosomes of the four inbred founders are represented by different colors and labeled as A, B, C, and D. The short bars denote Y chromosomes. The ancestral origin D is impossible in the X chromosomes of generation *t* ≥ 1. (B) Evaluation of the exchangeability assumption by one-locus genotype probabilities. The gray dashed line refers to the average genotype probability for one particular non-IBD genotype AB, AC, or BC; the black dashed line is for one particular IBD genotype AA, BB, or CC. Note that the ancestral origins A and B are exchangeable, but the ancestral origin C is not exchangeable with either A or B. (C) Schematics of the seven junction types along the maternally (left) and paternally (right) derived X chromosomes. (D) The rate matrix of the CTMC for the four RILs in (A). The diagonal elements are given so that row sums are zero. The rate matrix is determined by the seven basic rates, each corresponding to one of the seven junction types. The subscripts of the basic rates denote the IBD (1) or non-IBD (0) states on the left- and right-hand sides of the junctions, and the rates with superscript * refer to the transitions on the paternally derived chromosome. (E) The general relationships between the basic rates and the expected densities for the seven types of junctions, with L=3 for the four-way RIL in (A).

The ancestral origins along two homologous autosomes have been modeled as a continuous time Markov chain (CTMC) (Zheng *et al.* 2014). We extend the approach to account for the asymmetry of XX chromosomes, using superscript *m* (*p*) for maternally (paternally) derived genes or chromosomes. See Supporting Information, Table S1 for a list of symbols used in this paper. Let $O\left(x\right)=\left(O^{m}\left(x\right),O^{p}\left(x\right)\right)$ be the ordered pair of the ancestral origins at location *x* along the two X chromosomes of a randomly sampled female. The ancestral origin process O(x) is assumed to follow a CTMC, where *x* is the time parameter in unit of Morgan. We assign a unique ancestral origin to the X chromosomes of each inbred founder, or to each X chromosome of each outbred founder. Multiple genes, within or between loci, are identical by descent (IBD) if they have the same ancestral origins. Let *L* be the number of possible ancestral origins that $O^{m}\left(x\right)$ or $O^{p}\left(x\right)$ may take. *L* may be less than the number of inbred founders if some male founders did not produce daughters to pass down their X chromosomes. For example, L=3 for the four-way RIL by sibling mating since one of the founder mating pairs produces only one son (Figure 1A).

The *L* possible ancestral origins are assumed to be exchangeable, so that we focus on the changes of ancestral origins. See Figure 1B and the relevant part of Discussion on the exchangeability assumption. The initial distribution of O(0) at the leftmost locus x=0 is specified by $\alpha^{mp}\left(11\right)$, a probability that the two ancestral origins are the same (IBD) at a locus. Let $\alpha^{mp}\left(12\right)=1-\alpha^{mp}\left(11\right)$ be the non-IBD probability. Given either IBD or non-IBD at the locus, the ancestral origin pair O(0) takes each of the possible combinations with equal probability.

The transition rate matrix of the CTMC can be constructed from the expected densities $J^{mp}\left(abcd\right)$ of all the junction types (abcd) along the two X chromosomes of a female. The junction type (abcd) denotes the four-gene IBD configuration (abcd) on both sides of a junction, where ab (cd) is on the left-hand (right-hand) side, haplotype ac (bd) is on the first (second) chromosome, and the same integers denote IBD. Figure 1C illustrates the seven types of junctions: (1112), (1121), (1122), (1211), (1213), (1222), and (1232) for L≥3, where the two types (1213) and (1232) do not exist for L=2. We do not define junction types for the eight two-locus configurations (1111), (1123), (1212), (1221), (1223), (1231), (1233), and (1234), because there are either zero or no less than two junctions between the two loci. Figure 1D shows the transition rate matrix of the CTMC in the four-way RIL by sibling mating. Figure 1E shows the relationships between the expected densities $J^{mp}\left(abcd\right)$ and the transition rates, and they are derived based on the interpretation that $J^{mp}\left(abcd\right)\Delta d$ is the two-locus diplotype probability, in the limit that the genetic distance Δd (in Morgan) between two loci goes to zero.

The map expansions $R^{m}$ and $R^{p}$ and the overall expected junction density $\rho^{mp}$ are given by

$$R^{m}=J^{mp}\left(1121\right)+J^{mp}\left(1122\right)+J^{mp}\left(1222\right)+J^{mp}\left(1232\right),$$ (1)

$$R^{p}=J^{mp}\left(1112\right)+J^{mp}\left(1122\right)+J^{mp}\left(1211\right)+J^{mp}\left(1213\right),$$ (2)

$$\rho^{mp}=R^{m}+R^{p}-J^{mp}\left(1122\right),$$ (3)

similar to those for autosomes (Zheng *et al.* 2014) except that $R^{m}\neq R^{p}$ for X chromosomes. We have $J^{mp}\left(1112\right)=J^{mp}\left(1211\right)$ and $J^{mp}\left(1121\right)=J^{mp}\left(1222\right)$, since the junction densities do not depend on the direction of chromosomes. In contrast to the single-locus two-gene non-IBD probability $\alpha^{mp}\left(12\right)$, the ordering of the superscripts in $J^{mp}\left(abcd\right)$ generally does matter, that is, $J^{mp}\left(abcd\right)\neq J^{pm}\left(abcd\right)$ except for the junction type (1122). In addition, we have $J^{mp}\left(1213\right)=J^{pm}\left(1232\right)$ (see Figure 1C). Thus, the CTMC of X-linked ancestral origins can be described by one non-IBD probability $\alpha^{mp}\left(12\right)$ and the five expected junction densities $R^{m}$, $R^{p}$, $J^{mp}\left(1122\right)$, $J^{mp}\left(1232\right)$, and $J^{pm}\left(1232\right)$, under the exchangeability assumption of the *L* possible ancestral origins.

### Single-locus non-IBD probabilities

The calculation of the expected junction densities necessitates the introduction of the probabilities for the two- and three-gene IBD configurations at a single locus. All the following derivations of the recurrence relations for these probabilities are based on the Mendelian inheritance of X-linked genes: a paternally derived gene must be a copy of the maternally derived gene in a male of the previous generation, and a maternally derived gene has equal probability of being a copy of either the maternally derived gene or the paternally derived gene in a female of the previous generation.

**Figure 2:**
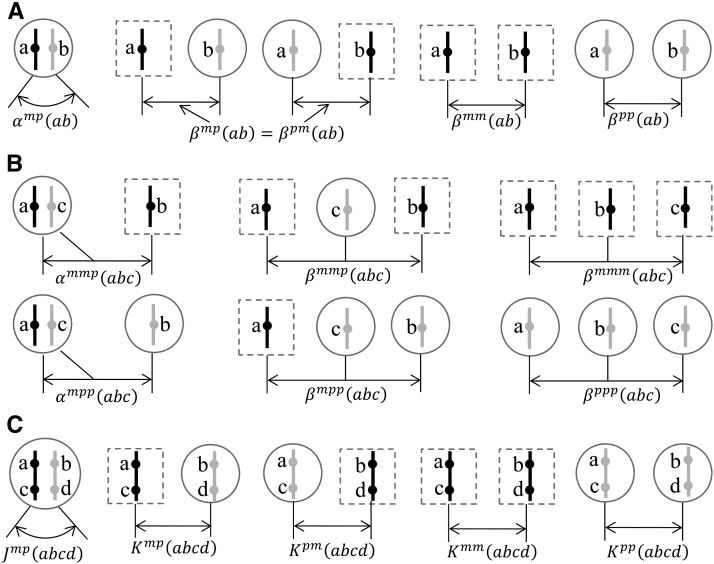
Schematics of (A) the probabilities of the two-gene IBD configurations, (B) the probabilities of the three-gene IBD configurations, and (C) the expected junction densities. Circles denote females, and dashed rectangles for males or females. Black vertical lines denote the maternally derived X chromosomes, and gray vertical lines for the paternally derived. Dots denote genes on chromosomes.

In a dioecious mapping population, the single-locus two-gene probabilities of IBD configuration (ab) depend on whether or not the two homologous genes are in a single individual. Thus, we denote by $\beta^{mm}\left(ab\right)$, $\beta^{mp}\left(ab\right)$, and $\beta^{pp}\left(ab\right)$ the two-gene probability of IBD configuration (ab), given that the two homologous genes are in two distinct individuals in generation *t* and have parental origins mm, mp, and pp, respectively (Figure 2A); it holds that $\beta^{pm}\left(ab\right)=\beta^{mp}\left(ab\right)$.

The recurrence relations of the two-gene non-IBD probabilities are derived by tracing the parental origins of two homologous genes from generation t≥1 into the previous generation, and they are given by

$$\beta_{t}^{mm}\left(12\right)=s_{t}^{m}\frac{1}{2}\alpha_{t-1}^{mp}\left(12\right)+\left(1-s_{t}^{m}\right)\left[\frac{1}{4}\beta_{t-1}^{mm}\left(12\right)+\frac{1}{2}\beta_{t-1}^{mp}+\frac{1}{4}\beta_{t-1}^{pp}\right]$$ (4a)

$$\beta_{t}^{mp}\left(12\right)=\frac{1}{2}\beta_{t-1}^{mm}\left(12\right)+\frac{1}{2}\beta_{t-1}^{mp}\left(12\right)$$ (4b)

$$\beta_{t}^{pp}\left(12\right)=\left(1-s_{t}^{p}\right)\beta_{t-1}^{mm}\left(12\right)$$ (4c)

$$\alpha_{t}^{mp}\left(12\right)=\beta_{t}^{mp}\left(12\right)$$ (4d)

where equation (4d) holds immediately after one generation of random mating, although it may not hold in the founder population at t=0. In equation (4a), the first term on the right-hand side refers to the scenario that the two genes with parent origins mm in generation *t* come from a single female of the previous generation with the probability $s_{t}^{m}$, and with probability 1/2 that they come from different genes of the female. In equation (4b), the two genes with parental origins mp cannot merge because they must come from one male and one female of the previous generation. In equation (4c), the two genes with parental origins pp in generation *t* come from a single male of the previous generation with the probability $s_{t}^{p}$; if so, they must merge because there is only one X chromosome in a male.

We introduce the single-locus three-gene probabilities of IBD configuration (abc). Let $\beta^{mmm}\left(abc\right)$, $\beta^{mmp}\left(abc\right)$, $\beta^{mpp}\left(abc\right)$, and $\beta^{ppp}\left(abc\right)$ be the probabilities of IBD configuration (abc), given that the three homologous genes are in three distinct individuals in generation *t* and have parental origins mmm, mmp, mpp, and ppp, respectively (Figure 2B). Similarly, we define $\alpha^{mmp}\left(abc\right)$ and $\alpha^{mpp}\left(abc\right)$ for three homologous genes in two distinct individuals. The ordering of the superscripts does not matter for these three-gene probabilities, for example, $\beta^{mmp}\left(abc\right)=\beta^{mpm}\left(abc\right)=\beta^{pmm}\left(abc\right)$.

The recurrence relations of the three-gene non-IBD probabilities are derived by tracing the parental origins of three homologous genes from generation *t* ≥ 1 into the previous generation, and they are given by

$$\begin{array}{c} \beta_{t}^{mmm}\left(123\right)=3q_{t}^{m}\frac{1}{2}\left[\frac{1}{2}\alpha_{t-1}^{mmp}\left(123\right)+\frac{1}{2}\alpha_{t-1}^{mpp}\left(123\right)\right]+\left(1-s_{t}^{m}-2q_{t}^{m}\right)\\ \times \left[\frac{1}{8}\beta_{t-1}^{mmm}\left(123\right)+\frac{3}{8}\beta_{t-1}^{mmp}\left(123\right)+\frac{3}{8}\beta_{t-1}^{mpp}\left(123\right)+\frac{1}{8}\beta_{t-1}^{ppp}\left(123\right)\right] \end{array}$$ (5a)

$$\begin{array}{c} \beta_{t}^{mmp}\left(123\right)=s_{t}^{m}\frac{1}{2}\alpha_{t-1}^{mmp}\left(123\right)+\left(1-s_{t}^{m}\right)\\ \times \left[\frac{1}{4}\beta_{t-1}^{mmm}\left(123\right)+\frac{1}{2}\beta_{t-1}^{mmp}\left(123\right)+\frac{1}{4}\beta_{t-1}^{mpp}\left(123\right)\right] \end{array}$$ (5b)

$$\beta_{t}^{mpp}\left(123\right)=\left(1-s_{t}^{p}\right)\left[\frac{1}{2}\beta_{t-1}^{mmm}\left(123\right)+\frac{1}{2}\beta_{t-1}^{mmp}\left(123\right)\right]$$ (5c)

$$\beta_{t}^{ppp}\left(123\right)=\left(1-s_{t}^{p}-2q_{t}^{p}\right)\beta_{t-1}^{mmm}\left(123\right)$$ (5d)

$$\alpha_{t}^{mmp}\left(123\right)=\beta_{t}^{mmp}\left(123\right)$$ (5e)

$$\alpha_{t}^{mpp}\left(123\right)=\beta_{t}^{mpp}\left(123\right)$$ (5f)

where $q_{t}^{m}$ is the coalescence probability of three maternally derived genes in generation *t* that a particular pair of genes come from a single female of the previous generation and the third comes from another female of the previous generation, and similarly $q_{t}^{p}$ for three paternally derived genes. The equations (5e, 5f) hold immediately after one generation of random mating, although they may not hold in the founder population at t=0.

The derivations of the recurrence equations (5a–5d) for the three-gene non-IBD probabilities are similar to equations (4a–4c) for the two-gene non-IBD probabilities. In equation (5a), the pre-factor 3 denotes that each of the three possible pairs of genes may come from a single female of the previous generation; the term $\left(1-s_{t}^{m}-2q_{t}^{m}\right)$ is the probability that the three maternally derived genes in generation *t* come from three distinct females of the previous generation, and it is obtained by the probability $1-s_{t}^{m}$ that one pair of genes come from two distinct females minus the probability $2q_{t}^{m}$ that the third gene and either gene of the pair come from a single female of the previous generation. Similarly, the term $\left(1-s_{t}^{p}-2q_{t}^{p}\right)$ in equation (5d) is the probability that the three paternally derived genes in generation *t* come from three distinct males of the previous generation.

### Expected junction densities

We derive the recurrence relations for $R^{m}$, $R^{p}$, $J^{mp}\left(1122\right)$, $J^{mp}\left(1232\right)$, and $J^{pm}\left(1232\right)$. The recurrence relation for $R^{m}$ follows from the theory of junctions (Fisher 1954): a new junction is formed whenever a recombination event occurs between two X chromosomes that are non-IBD at the location of a crossover. The recurrence relations for the map expansions $R^{m}$ and $R^{p}$ are given by

$$R_{t}^{m}=\frac{1}{2}R_{t-1}^{m}+\frac{1}{2}R_{t-1}^{p}+\alpha_{t-1}^{mp}\left(12\right),$$ (6a)

$$R_{t}^{p}=R_{t-1}^{m},$$ (6b)

where equation (6b) follows directly from no recombination events occurring between the XY chromosomes in a male of the previous generation.

To measure differential map expansions between maternally and paternally derived chromosomes, we define $R_{t}^{X}=\left(2R_{t}^{m}+R_{t}^{p}\right)/3$ and $R_{t}^{-}=\left(R_{t}^{m}-R_{t}^{p}\right)/2$, and their recurrence relations are given by

$$R_{t}^{X}=R_{t-1}^{X}+\frac{2}{3}\alpha_{t-1}^{mp}\left(12\right),$$ (7a)

$$R_{t}^{-}=-\frac{1}{2}R_{t-1}^{-}+\frac{1}{2}\alpha_{t-1}^{mp}\left(12\right),$$ (7b)

according to the recurrence equations (6a, 6b). If there are equal numbers of males and females in the population, a randomly chosen X chromosome is maternally derived with probability 2/3, and it is paternally derived with probability 1/3. Thus $R_{t}^{X}$ can be interpreted as the map expansion on a randomly chosen X chromosome.

For comparisons, we denote by $R_{t}^{A}$ the map expansion on a random chosen autosome, and and its recurrence relation is given by (MacLeod *et al.* 2005; Zheng *et al.* 2014)

$$R_{t}^{A}=R_{t-1}^{A}+\alpha_{t-1}^{AA}\left(12\right),$$ (8)

where $\alpha_{t}^{AA}\left(12\right)$ refers to the non-IBD probability between two homologous autosomal genes in an individual. The equations (7a, 8) show that the map expansion $R_{t}^{X}$ for an X chromosome is two-thirds $R_{t}^{A}$ for an autosome if the non-IBD probability $\alpha_{t}^{AA}\left(12\right)$ for autosomes is the same as $\alpha_{t}^{mp}\left(12\right)$ for XX chromosomes, and the sex ratio is 1.

In addition to $J_{t}^{mp}\left(abcd\right)$ and $J_{t}^{pm}\left(abcd\right)$, we define $K_{t}^{mm}\left(abcd\right)$, $K_{t}^{mp}\left(abcd\right)$, $K_{t}^{pm}\left(abcd\right)$, and $K_{t}^{pp}\left(abcd\right)$ for haplotypes ac and bd that are in two distinct individuals and have parental origins mm, mp, pm, and pp, respectively (Figure 2C). The contributions to the junctions in the current generation come from either the existing junctions at the previous generation, or a new junction via a crossover event. In the following, we focus on the formation of a new junction, because the contributions of the existing junctions in the previous generation are similar to those for the two-gene non-IBD probabilities in the recurrence equations (4a–4c).

The schematics of the recurrence relations for junction types (1232) and (1122) are shown in Figure S1. The ancestry transitions of type (1122) occur on both haplotypes ac and bd at exactly the same location, and thus a new junction of type (1122) can be formed only by duplicating a chromosome segment. It holds that $J_{t}^{mp}\left(1122\right)=J_{t}^{pm}\left(1122\right)$ and $K_{t}^{mp}\left(1122\right)=K_{t}^{pm}\left(1122\right)$ because of the symmetry of type (1122). We have

$$\begin{array}{c} K_{t}^{mm}\left(1122\right)=s_{t}^{m}\left[\frac{1}{2}J_{t-1}^{mp}\left(1122\right)+\frac{1}{4}R_{t-1}^{m}+\frac{1}{4}R_{t-1}^{p}\right]\\ +\left(1-s_{t}^{m}\right)\left[\frac{1}{4}K_{t-1}^{mm}\left(1122\right)+\frac{1}{2}K_{t-1}^{mp}\left(1122\right)+\frac{1}{4}K_{t-1}^{pp}\left(1122\right)\right], \end{array}$$ (9a)

$$K_{t}^{mp}\left(1122\right)=\frac{1}{2}K_{t-1}^{mm}\left(1122\right)+\frac{1}{2}K_{t-1}^{mp}\left(1122\right),$$ (9b)

$$K_{t}^{pp}\left(1122\right)=s_{t}^{p}R_{t-1}^{m}+\left(1-s_{t}^{p}\right)K_{t-1}^{mm}\left(1122\right),$$ (9c)

$$J_{t}^{mp}\left(1122\right)=K_{t}^{mp}\left(1122\right),$$ (9d)

for *t* ≥ 1, where equation (9d) may not hold in the founder population at t=0, the first term on the right-hand side of equation (9a) refers to the scenario that both haplotypes ac and bd come from a single female of the previous generation, and the first term on the right-hand side of equation (9c) refers to the scenario that both haplotypes are the duplicated copies of the maternally derived X chromosome in a male of the previous generation (Figure S1A). According to equations (6a, 6b) and equations (9a–9d), the overall expected density $\rho^{mp}$ in equation (3) does not depend on the three-gene non-IBD probabilities.

The ancestry transition of type (1232) occurs on haplotype ac. A new junction of type (1232) is formed whenever the two parental chromosomes of haplotype ac and the parental chromosome of haplotype bd are distinct and have the IBD configuration (123) at the location of the crossover. We have

$$\begin{array}{c} K_{t}^{mm}\left(1232\right)=s_{t}^{m}\left[\frac{1}{4}J_{t-1}^{mp}\left(1232\right)+\frac{1}{4}J_{t-1}^{pm}\left(1232\right)\right]\\ +\left(1-s_{t}^{m}\right)\left\{\frac{1}{4}\left[K_{t-1}^{mm}\left(1232\right)+\alpha_{t-1}^{mmp}\left(123\right)\right]+\frac{1}{4}\left[K_{t-1}^{mp}\left(1232\right)+\alpha_{t-1}^{mpp}\left(123\right)\right]\right\}\\ +\left(1-s_{t}^{m}\right)\left\{\frac{1}{4}\left[K_{t-1}^{pm}\left(1232\right)+\alpha_{t-1}^{mmp}\left(123\right)\right]+\frac{1}{4}\left[K_{t-1}^{pp}\left(1232\right)+\alpha_{t-1}^{mpp}\left(123\right)\right]\right\}, \end{array}$$ (10a)

$$K_{t}^{mp}\left(1232\right)=\frac{1}{2}\left[K_{t-1}^{mm}\left(1232\right)+\alpha_{t-1}^{mmp}\left(123\right)\right]+\frac{1}{2}\left[K_{t-1}^{pm}\left(1232\right)+\alpha_{t-1}^{mmp}\left(123\right)\right],$$ (10b)

$$K_{t}^{pm}\left(1232\right)=\frac{1}{2}K_{t-1}^{mm}\left(1232\right)+\frac{1}{2}K_{t-1}^{mp}\left(1232\right),$$ (10c)

$$K_{t}^{pp}\left(1232\right)=\left(1-s_{t}^{p}\right)K_{t-1}^{mm}\left(1232\right),$$ (10d)

$$J_{t}^{mp}\left(1232\right)=K_{t}^{mp}\left(1232\right),$$ (10e)

$$J_{t}^{pm}\left(1232\right)=K_{t}^{pm}\left(1232\right),$$ (10f)

for t≥1, where equations (10e, 10f) may not hold in the founder population at t=0. A new junction is formed at the rate $\alpha_{t-1}^{mmp}\left(123\right)$ ($\alpha_{t-1}^{mpp}\left(123\right)$), given that the parental chromosome of haplotype bd is maternally (paternally) derived. The density $K_{t}^{pm}\left(1232\right)$ in equation (10c) has no contributions of a new junction because there are no crossover events occurring between the XY chromosomes in the father of haplotype ac (Figure S1B). We denote by $K_{t}^{mp+}\left(1232\right)=\left[K_{t}^{mp}\left(1232\right)+K_{t}^{pm}\left(1232\right)\right]/2$, and $K_{t}^{mp-}\left(1232\right)=\left[K_{t}^{mp}\left(1232\right)-K_{t}^{pm}\left(1232\right)\right]/2$, and their recurrence relations are given in Appendix A. Both $K_{t}^{mp-}\left(1232\right)$ and $R_{t}^{-}$ measure the asymmetry between maternally and paternally derived X chromosomes.

### Model evaluation by simulations

To evaluate the theoretical predications of non-IBD probabilities and expected junction densities, we perform simulation studies with the same model assumptions: random mating with discrete generations, no natural selections, and no genetic interferences, except that the ancestral origins along chromosomes do not follow Marker assumptions. Instead, the genome ancestral origins are simulated forwardly by first generating a pedigree according to a given breeding design, and then dropping on the pedigree the distinct founder genome labels (ancestral origins) that are assigned to the whole X chromosomes of each complete inbred founder or to each X chromosome of each outbred founder. The X chromosomes of each descendant gamete are specified as a list of the labeled segments determined by chromosomal crossovers.

For a mapping population with the particular breeding design, the realized junction densities and IBD probabilities are saved for all individuals in each generation in each simulation replicate, and they are averaged over in total $2\times 10^{4}$ replicates. Various mating schemes are used in simulating breeding pedigrees. We denote by RM1 the random mating where each sampling of two randomly chosen individuals with opposite genders produces *one* offspring, and RM2 the random mating where each sampling of two randomly chosen individuals with opposite genders produces *two* offspring. We combine these mating schemes with -NE if each parent contributes a Poisson distributed number of gametes to the next generation, and -E if each parent contributes exactly two gametes. Thus, we have four random mating schemes, RM1-NE, RM1-E, RM2-NE, and RM2-E. The sibling mating belongs to RM2-E with population size 2, and the exclusively pairing in $2^{n}$-way (*n* ≥ 1) crosses can be regarded as a special case of random mating without inbreeding. The genders are assigned randomly, independent of mating schemes.

## Application to QTL mapping populations

### Multistage populations

For mapping populations with stage-wise constant mating schemes, we derive analytic expressions of the non-IBD probabilities and the expected junction densities for constructing CTMC of X-linked ancestral origins, according to the recurrence relations. The closed form solutions are obtained by linking results of each subsequent stage via the initial conditions. The general results for a population with constant random mating are derived in Appendix A, where three scenarios are considered: finite population of size ≥6, sibling-mating population of size 2, and large population of size »6. Table S2 gives the coalescence probabilities of X chromosomes for various mating schemes, similar to Table 1 of Zheng *et al.* (2014) for autosomes. Table S3 summarizes the results for X chromosomes in a sibling-mating population, and Table S4 for autosomes; they are necessary for dioecious breeding populations with a stage of inbreeding by sibling mating such as the CC and the DSPR. We use the superscripts of *A* denoting the quantities for autosomes.

**Table 1:** Results for X chromosomes in the $2^{n}$-way RIL by sibling mating in the last generation g=U+V+1, where U=0 for n=1 and U=n−2 for n≥2

Quantity	Theoretical Prediction
(A) 2 ways sibling	
$\alpha_{g}^{mp}\left(12\right)$	$\frac{5+\sqrt{5}}{10}{\left(\lambda_{1}\right)}^{V}+conjugate$
$R_{g}^{X}$	$\frac{8}{3}-\frac{20+8\sqrt{5}}{15}{\left(\lambda_{1}\right)}^{V}+conjugate$
$R_{g}^{m}$	$\frac{8}{3}-\frac{2}{3}{\left(-\frac{1}{2}\right)}^{V}-\frac{5+3\sqrt{5}}{5}{\left(\lambda_{1}\right)}^{V}+conjugate$
$J_{g}^{mp}\left(1122\right)$	$\frac{8}{3}+\frac{1}{3}{\left(-\frac{1}{2}\right)}^{V}-\left(\frac{3+\sqrt{5}}{2}+\frac{5+\sqrt{5}}{10}V\right){\left(\lambda_{1}\right)}^{V}+conjugate$
$J_{g}^{mp+}\left(1232\right)$	0
$J_{g}^{mp-}\left(1232\right)$	0
(B) $2^{n}\left(n\geq 2\right)$ ways sibling	
$\alpha_{g}^{mp}\left(12\right)$	$\frac{5+3\sqrt{5}}{10}{\left(\lambda_{1}\right)}^{V}+conjugate$
$R_{g}^{X}$	$\frac{2}{3}\left(U+6\right)-\frac{30+14\sqrt{5}}{15}{\left(\lambda_{1}\right)}^{V}+conjugate$
$R_{g}^{m}$	$\frac{2}{3}\left(U+6\right)+\frac{1}{3}\left(R_{U+1}^{m}-R_{U+1}^{p}\right){\left(-\frac{1}{2}\right)}^{V}-\frac{10+4\sqrt{5}}{5}{\left(\lambda_{1}\right)}^{V}+conjugate$
$J_{g}^{mp}\left(1122\right)$	$\begin{array}{l} \frac{2}{3}\left(U+6\right)-\frac{1}{6}\left(R_{U+1}^{m}-R_{U+1}^{p}\right){\left(-\frac{1}{2}\right)}^{V}\\ -\left[\frac{10+4\sqrt{5}}{5}+\frac{1+\sqrt{5}}{4}R_{U+1}^{m}+\frac{5+\sqrt{5}}{20}R_{U+1}^{p}+\frac{5+3\sqrt{5}}{10}V\right]{\left(\lambda_{1}\right)}^{V}+conjugate \end{array}$
$J_{g}^{mp+}\left(1232\right)$	$-{\left(\frac{1}{2}\right)}^{V}+\left[\frac{5+3\sqrt{5}}{10}+\frac{1+\sqrt{5}}{4}R_{U+1}^{m}+\frac{5+\sqrt{5}}{20}R_{U+1}^{p}\right]{\left(\lambda_{1}\right)}^{V}+conjugate$
$J_{g}^{mp-}\left(1232\right)$	${\left(\frac{1}{2}\right)}^{V+1}+\left(R_{U+1}^{p}-R_{U+1}^{m}+1\right){\left(-\frac{1}{2}\right)}^{V+1}$

The eigenvalues $\lambda_{1}=(1+\sqrt{5})/4$ and $\lambda_{2}=(1-\sqrt{5})/4$. The map expansions $R_{U+1}^{p}$ and $R_{U+1}^{m}$ are given by equations (11a, 11b). The conjugate is given by replacing $\sqrt{5}$ with $-\sqrt{5}$ from the terms involving $\lambda_{1}$. For example, the conjugate term for $R_{g}^{X}$ in (A) is given by $-(20-8\surd 5){\left(\lambda_{2}\right)}^{V}/15\;$. RIL, recombinant inbred line.

We derive the analytic expressions of $\alpha_{t}^{mp}\left(12\right)$, $R_{t}^{X}$, $R_{t}^{m}$, $J_{t}^{mp}\left(1122\right)$, $J_{t}^{mp+}\left(1232\right)$, and $J_{t}^{mp-}\left(1232\right)$ in the mapping populations of the RIL, the AIL, and the DO, and they are given in Table 1, Table 2, and Table 3, respectively. These results are necessary for constructing the CTMC of ancestral origins along the XX chromosomes of a female; only the expression of $R_{t}^{m}$ is needed for the maternal derived X chromosome of a male. For comparisons, the autosomal results for $\alpha_{t}^{AA}\left(12\right)$, $R_{t}^{A}$, $J_{t}^{AA}\left(1122\right)$, and $J_{t}^{AA}\left(1232\right)$ are included. The results for the AIL, the DO, and the DSPR are derived under the assumption of a large population size in the intercross stage. We evaluate this assumption in the DSPR, because the evaluation results hold similarly for the AIL and the DO. In addition, the map expansions $R_{t}^{X}$ and $R_{t}^{A}$ are given explicitly under the assumption of an infinitely large intercross population size, which may be used as a simple measure of QTL mapping resolution.

**Table 2:** Results for the AIL in the last generation g=U+1

Quantity	Theoretical Prediction
(A) X chromosomes	
$\alpha_{g}^{mp}(12)$	$\left(1-\frac{10}{9L}\right){\left(\lambda_{1}\right)}^{U}+\frac{2}{9L}{\left(-\frac{1}{2}\right)}^{U}+\frac{8}{9L}{\left(\frac{1}{4}\right)}^{U}$
$R_{g}^{X}$	$\frac{8}{9L}+\frac{2}{3}\left(1-\frac{10}{9L}\right)\frac{1-{\left(\lambda_{1}\right)}^{U}}{1-\lambda_{1}}-\frac{8}{81L}{\left(-\frac{1}{2}\right)}^{U}-\frac{64}{81L}{\left(\frac{1}{4}\right)}^{U}$
$R_{g}^{m}$	$\frac{2}{9}+\frac{52}{81L}+\frac{2}{3}\left(1-\frac{10}{9L}\right)\frac{1-{\left(\lambda_{1}\right)}^{U}}{1-\lambda_{1}}-\left(\frac{2}{9}+\frac{20}{81L}+\frac{4}{27L}U\right){\left(-\frac{1}{2}\right)}^{U}-\frac{32}{81L}{\left(\frac{1}{4}\right)}^{U}$
$J_{g}^{mp}\left(1122\right)$	$\frac{2}{9}+\frac{52}{81L}+\frac{2}{3}\left(1-\frac{10}{9L}\right)\frac{1-{\left(\lambda_{1}\right)}^{U}}{1-\lambda_{1}}-\left[\frac{2}{9}+\frac{52}{81L}+\left(\frac{2}{3}+\frac{16}{27}s\right)\left(1-\frac{10}{9L}\right)U\right]{\left(\lambda_{1}\right)}^{U}$
$J_{g}^{mp+}\left(1232\right)$	$\left(1-\frac{2}{L}\right)\left(1-\frac{4}{3L}\right)\frac{{\left(\lambda_{1}\right)}^{U}-{\left(\lambda_{4}\right)}^{U}}{s}+\left(1-\frac{2}{L}\right)\left(-\frac{1}{9}+\frac{76}{81L}\right){\left(\lambda_{1}\right)}^{U}+\left(1-\frac{2}{L}\right)\frac{16}{81L}{\left(\lambda_{4}\right)}^{U}$$+\left(1-\frac{2}{L}\right)\left(\frac{1}{9}-\frac{4}{81L}+\frac{8}{27L}U\right){\left(-\frac{1}{2}\right)}^{U}+\left(1-\frac{2}{L}\right)\left(-\frac{88}{81L}+\frac{16}{27L}U\right){\left(\frac{1}{4}\right)}^{U}$
$J_{g}^{mp-}\left(1232\right)$	$\left(1-\frac{2}{L}\right)\left(\frac{1}{3}-\frac{4}{9L}\right){\left(\lambda_{4}\right)}^{U}-\left(1-\frac{2}{L}\right)\left(\frac{1}{3}+\frac{4}{9L}\right){\left(-\frac{1}{2}\right)}^{U}+\left(1-\frac{2}{L}\right)\frac{8}{9L}{\left(\frac{1}{4}\right)}^{U}$
(B) Autosomes	
$\alpha_{g}^{AA}(12)$	$\left(1-\frac{1}{L}\right){\left(\lambda_{1}^{A}\right)}^{U-1}$
$R_{g}^{A}$	$1+\left(1-\frac{1}{L}\right)\frac{1-{\left(\lambda_{1}^{A}\right)}^{U-1}}{1-\lambda_{1}^{A}}$
$J_{g}^{AA}\left(1122\right)$	$\left[1-{\left(\lambda_{1}^{A}\right)}^{U-1}\right]+\left(1-\frac{1}{L}\right)\left[\frac{1-{\left(\lambda_{1}^{A}\right)}^{U-1}}{1-\lambda_{1}^{A}}-\left(U-1\right){\left(\lambda_{1}^{A}\right)}^{U-2}\right]$
$J_{g}^{AA}\left(1232\right)$	$\left(1-\frac{2}{L}\right){\left(\lambda_{1}^{A}\right)}^{U-1}+\left(1-\frac{1}{L}\right)\left(1-\frac{2}{L}\right)\frac{{\left(\lambda_{1}^{A}\right)}^{U-1}-{\left(\lambda_{4}^{A}\right)}^{U-1}}{s^{A}}$

The eigenvalues $\lambda_{1}=1-s/3$ and $\lambda_{4}=1-s$ for X chromosomes, and for autosomes $\lambda_{1}^{A}=1-s^{A}/2$ and $\lambda_{4}^{A}=1-3s^{A}/2$. AIL, advanced inter-cross lines.

**Table 3:** Results for the DO in the last generation g=U+1

Quantity	Theoretical Prediction
(A) X chromosomes	
$\alpha_{g}^{mp}(12)$	$\left(1-\frac{1}{L}\right){\left(\lambda_{1}\right)}^{U}$
$R_{g}^{X}$	$R_{0}^{X}+\frac{2}{3}\alpha_{0}^{mp}\left(12\right)+\frac{2}{3}\left(1-\frac{1}{L}\right)\frac{1-{\left(\lambda_{1}\right)}^{U}}{1-\lambda_{1}}$
$R_{g}^{m}$	$\begin{array}{l} R_{0}^{X}+\frac{2}{3}\alpha_{0}^{mp}\left(12\right)+\frac{2}{9}\left(1-\frac{1}{L}\right)+\frac{2}{3}\left(1-\frac{1}{L}\right)\frac{1-{\left(\lambda_{1}\right)}^{U}}{1-\lambda_{1}}\\ -\left[\frac{2}{9}\left(1-\frac{1}{L}\right)+\frac{1}{6}\left(R_{0}^{m}-R_{0}^{p}\right)-\frac{1}{3}\alpha_{0}^{mp}\left(12\right)\right]{\left(-\frac{1}{2}\right)}^{U} \end{array}$
$J_{g}^{mp}\left(1122\right)$	$\begin{array}{l} R_{0}^{X}+\frac{2}{3}\alpha_{0}^{mp}\left(12\right)+\frac{2}{9}\left(1-\frac{1}{L}\right)+\frac{2}{3}\left(1-\frac{1}{L}\right)\frac{1-{\left(\lambda_{1}\right)}^{U}}{1-\lambda_{1}}\\ -\left[R_{0}^{X}+\frac{2}{3}\alpha_{0}^{mp}\left(12\right)+\frac{2}{9}\left(1-\frac{1}{L}\right)+\left(\frac{2}{3}+\frac{16}{27}s\right)\left(1-\frac{1}{L}\right)U\right]{\left(\lambda_{1}\right)}^{U} \end{array}$
$J_{g}^{mp+}\left(1232\right)$	$\begin{array}{l} \left(1-\frac{1}{L}\right)\left(1-\frac{2}{L}\right)\frac{{\left(\lambda_{1}\right)}^{U}-{\left(\lambda_{4}\right)}^{U}}{s}+\left(1-\frac{2}{L}\right)\left[-\frac{1}{9}\left(1-\frac{1}{L}\right)+R_{0}^{X}+\frac{2}{3}\alpha_{0}^{mp}\left(12\right)\right]{\left(\lambda_{1}\right)}^{U}\\ +\left(1-\frac{2}{L}\right)\left[\frac{1}{9}\left(1-\frac{1}{L}\right)+\frac{1}{12}\left(R_{0}^{m}-R_{0}^{p}\right)-\frac{1}{6}\alpha_{0}^{mp}\left(12\right)\right]{\left(-\frac{1}{2}\right)}^{U} \end{array}$
$J_{g}^{mp-}\left(1232\right)$	$\frac{1}{3}\left(1-\frac{1}{L}\right)\left(1-\frac{2}{L}\right){\left(\lambda_{4}\right)}^{U}+\left(1-\frac{2}{L}\right)\left[-\frac{1}{3}\left(1-\frac{1}{L}\right)-\frac{1}{4}\left(R_{0}^{m}-R_{0}^{p}\right)+\frac{1}{2}\alpha_{0}^{mp}\left(12\right)\right]{\left(-\frac{1}{2}\right)}^{U}$
(B) Autosomes	
$\alpha_{g}^{AA}(12)$	$\left(1-\frac{1}{L}\right){\left(\lambda_{1}^{A}\right)}^{U}$
$R_{g}^{A}$	$R_{0}^{A}+\alpha_{0}^{AA}\left(12\right)+\left(1-\frac{1}{L}\right)\frac{1-{\left(\lambda_{1}^{A}\right)}^{U}}{1-\lambda_{1}^{A}}$
$J_{g}^{AA}\left(1122\right)$	$\left[R_{0}^{A}+\alpha_{0}^{AA}\left(12\right)\right]\left[1-{\left(\lambda_{1}^{A}\right)}^{U}\right]+\left(1-\frac{1}{L}\right)\left[\frac{1-{\left(\lambda_{1}^{A}\right)}^{U}}{1-\lambda_{1}^{A}}-U{\left(\lambda_{1}^{A}\right)}^{U-1}\right]$
$J_{g}^{AA}\left(1232\right)$	$\left[R_{0}^{A}+\alpha_{0}^{AA}\left(12\right)\right]\left(1-\frac{2}{L}\right){\left(\lambda_{1}^{A}\right)}^{U}+\left(1-\frac{1}{L}\right)\left(1-\frac{2}{L}\right)\frac{{\left(\lambda_{1}^{A}\right)}^{U}-{\left(\lambda_{4}^{A}\right)}^{U}}{s^{A}}$

The eigenvalues $\lambda_{1}=1-s/3$ and $\lambda_{4}=1-s$ for X chromosomes, and for autosomes $\lambda_{1}^{A}=1-s^{A}/2$ and $\lambda_{4}^{A}=1-3s^{A}/2$. DO, diversity outcross.

Many breeding populations can be divided into three stages: mixing, intercross, and inbreeding, such as the RIL by sibling mating, the CC, and the DSPR. There is no inbreeding stage for the AIL, the HS, and the DO. We denote by *U* the number of intercross generations, *V* the number of inbreeding generations, and *N* the intercross population size. Let ${ℳ}_{F}$ and ${ℳ}_{I}$ denote the random mating schemes for mixing and intercross stages, respectively. We choose the mixing stage to consist of one generation of random mating, so that the non-IBD probabilities and the expected junction densities in the $F_{1}$ population do not depend on whether genes or haplotypes are in distinct individuals.

The general derivation procedure is as follows. First, we derive the initial conditions in the $F_{1}$ population for the intercross stage, according to the genetic compositions of the founder population $F_{0}$. Second, we substitute the obtained initial conditions into the theorems of Appendix A3 under the assumption of a large intercross population size. Alternatively, the theorems of Appendix A1 may be used for a finite intercross population. Lastly, if there is a stage of inbreeding by sibling mating, we substitute analytic expressions in the $F_{U+1}$ population into the theorems of Appendix A2 to obtain the results in the last generation g=U+V+1.

### RIL

The $2^{n}$-way RIL by sibling mating can be regarded as a three-stage mapping population without the intercross stage for n≤2. All the founders are fully inbred, and the intercross mating scheme is exclusively pairing so that inbreeding is completely avoided. Thus $R_{1}^{m}=R_{1}^{p}=0$, and the non-IBD probability $\alpha_{t}^{mp}\left(12\right)=1$ during the intercross stage 1≤t≤U+1, where U=0 for n=1 and U=n−2 for n≥2. According to the recurrence equations (6a, 6b), it holds

$$R_{U+1}^{m}=\frac{2}{9}\left[1+3U-{\left(-\frac{1}{2}\right)}^{U}\right],$$ (11a)

$$R_{U+1}^{p}=R_{U}^{m},$$ (11b)

and $R_{U+1}^{X}=2U/3$. Furthermore, it is straightforward to obtain $\beta_{U+1}^{mp}\left(12\right)=1$, $\beta_{U+1}^{mm}\left(12\right)=\alpha_{U+1}^{mmp}\left(123\right)=\delta_{n\geq 2}$, $K_{U+1}^{mm}\left(1122\right)=K_{U+1}^{mp}\left(1122\right)=0$, $K_{U+1}^{mm}\left(1232\right)=K_{U+1}^{mp}\left(1232\right)=R_{U+1}^{m}$, and $K_{U+1}^{pm}\left(1232\right)=R_{U+1}^{p}$, where the indicator $\delta_{n\geq 2}=1$ if n≥2 and 0 otherwise, since the two maternally derived genes at t=1 must come from the inbred female founder for the two-way RIL.

Substituting the initial conditions in the $F_{U+1}$ population into Table S3, we obtain the results for the RIL in the last generation t=U+V+1 shown in Table 1. The non-IBD probabilities $\alpha_{t}^{mp}\left(12\right)$ for X chromosomes are the same as those for autosomes (Table 2 of Zheng *et al.* 2014). Thus, we show analytically that the map expansion $R^{X}$ for the X chromosome is two-thirds that of the autosome for the $2^{n}$-way (*n* ≥ 1) RIL, according to equations (7a, 8). Broman (2012a) has verified this two-thirds rule via Maxima for the $2^{n}$-way RIL up to n=98.

**Figure 3:**
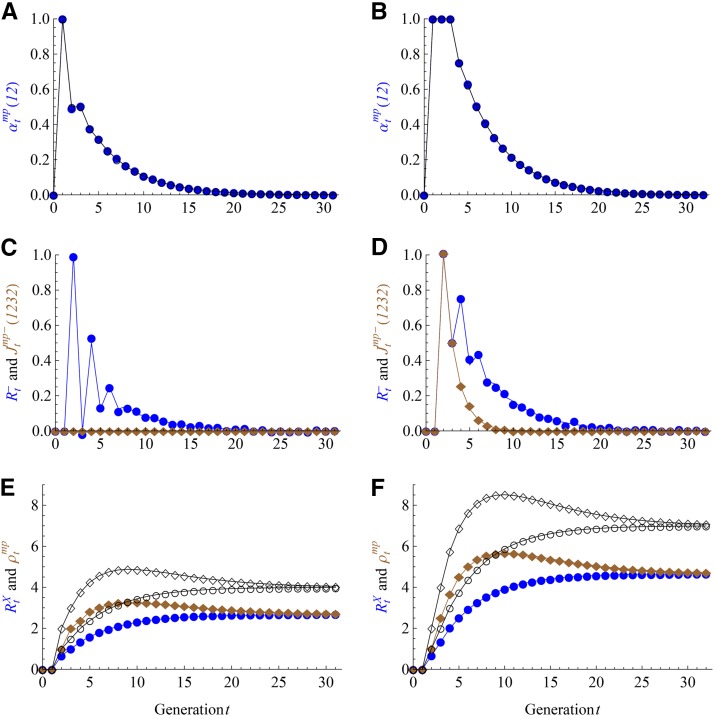
Results of the $2^{n}$-way recombinant inbred lines (RILs) with by sibling mating for n=1 (left panels) and n=3 (right panels). The filled symbols refer to the results for X chromosomes, the empty symbols for autosomes, and lines for the theoretical predictions in Table 1. The non-IBD probabilities $\alpha_{t}^{mp}\left(12\right)$ for X chromosomes and autosomes are overlapped with each other. The brown filled diamonds refer to $J_{t}^{mp-}\left(1232\right)$ in (C) and (D) and $\rho_{t}^{mp}$ in (E) and (F).

Figure 3 shows that these theoretical predictions fit very well with the forward simulation results for the two- and eight-way RIL by sibling mating. The differential densities $R_{t}^{-}$ and $J_{t}^{mp-}\left(1232\right)$ decay very fast with generation *t* and show some oscillations in the beginning generations. The overall expected junction density $\rho_{t}^{mp}$ reaches the maximum in the same generation for autosomes.

### AIL

We consider a multiparental AIL population that is founded by L/2 inbred females and L/2 inbred males. A unique ancestral origin is assigned to each inbred founder’s genomes so that the two-gene non-IBD probabilities $\alpha_{0}^{mp}\left(12\right)=0$ and $\beta_{0}^{mm}\left(12\right)=\beta_{0}^{mp}\left(12\right)=\beta_{0}^{pp}\left(12\right)=1$, and similarly for the three-gene non-IBD probabilities $\alpha_{0}^{mmp}\left(123\right)=\alpha_{0}^{mpp}\left(123\right)=0$, $\beta_{0}^{mmm}\left(123\right)=\beta_{0}^{mmp}\left(123\right)=\beta_{0}^{mpp}\left(123\right)=\beta_{0}^{ppp}\left(123\right)=1$ if they exist.

The $F_{1}$ population of size *N* is produced by mating scheme ${ℳ}_{F}=$ RM1-NE or RM2-NE. According to Table S2, the coalescence probabilities $s_{1}^{m}=s_{1}^{p}=2/L$ and $q_{1}^{m}=q_{1}^{p}=\left(2/L\right)\left(1-2/L\right)$ for mating scheme RM1-NE, and they hold approximately for RM2-NE with large population size *N* » 6. Thus, the two-gene non-IBD probabilities at t=1 are given by $\beta_{1}^{mm}\left(12\right)=\beta_{1}^{pp}\left(12\right)=1-2/L$ and $\alpha_{1}^{mp}\left(12\right)=\beta_{1}^{mp}\left(12\right)=1$ according to the recurrence equations (4a–4d), and the three-gene non-IBD probabilites at t=1 are given by $\beta_{1}^{mmm}\left(123\right)=\beta_{1}^{ppp}\left(123\right)=\left(1-2/L\right)\left(1-4/L\right)$ and $\alpha_{1}^{mmp}\left(123\right)=\alpha_{1}^{mpp}\left(123\right)=\beta_{1}^{mmp}\left(123\right)=\beta_{1}^{mpp}\left(123\right)=1-2/L$ according to the recurrence equations (5a–5f). In addition, no junctions can be formed from inbred founders so that it holds that $R_{1}^{m}=R_{1}^{p}=0$, $K_{1}^{mm}\left(1122\right)=K_{1}^{mp}\left(1122\right)=K_{1}^{pp}\left(1122\right)=0$, and $K_{1}^{mm}\left(1232\right)=K_{1}^{mp}\left(1232\right)=K_{1}^{pm}\left(1232\right)=K_{1}^{pp}\left(1232\right)=0$.

The $F_{1}$ population is maintained for *U* generations with constant size *N* and sex ratio 1. Assuming that the intercross population size is large (*N* » 6), all the two- and three-gene coalescence probabilities at t≥2 are approximately equal and are denoted by *s*, and they are determined by the intercross mating scheme ${ℳ}_{I}$ according to Table S2. Substituting the initial conditions in the $F_{1}$ population into the theorems of Appendix A3, we obtain in Table 2 the results for X chromosomes in the AIL in the last generation t=U+1. Table 2 also shows the results for autosomes, which are derived according to Zheng *et al.* (2014).

As shown in Table 2, the non-IBD probabilities $\alpha_{t}^{mp}\left(12\right)$ for X-chromosomes are unequal to those for autosomes, and thus the map expansions generally do not satisfy the two-thirds rule. According to the map expansions $R_{t}^{X}$ and $R_{t}^{A}$ in Table 2, we derive their approximations under the limit of an infinitely large population size (*N* →∞) so that the coalescence probability goes to zero (*s* →0),

$$R_{U+1}^{X}\approx \frac{8}{9L}+\frac{2}{3}\left(1-\frac{10}{9L}\right)U,$$ (12a)

$$R_{U+1}^{A}\approx 1+\left(1-\frac{1}{L}\right)\left(U-1\right),$$ (12b)

where the last two terms for $R_{t}^{X}$ in Table 2 are small and thus ignored. The equations (12a, 12b) show that the two-thirds rule is approximately valid for a large number *L* of founder lines. The map expansion of equation (12b) for L=2 is consistent with the previous results (Darvasi and Soller 1995; Liu *et al.* 1996; Winkler *et al.* 2003; Broman 2012b).

**Figure 4:**
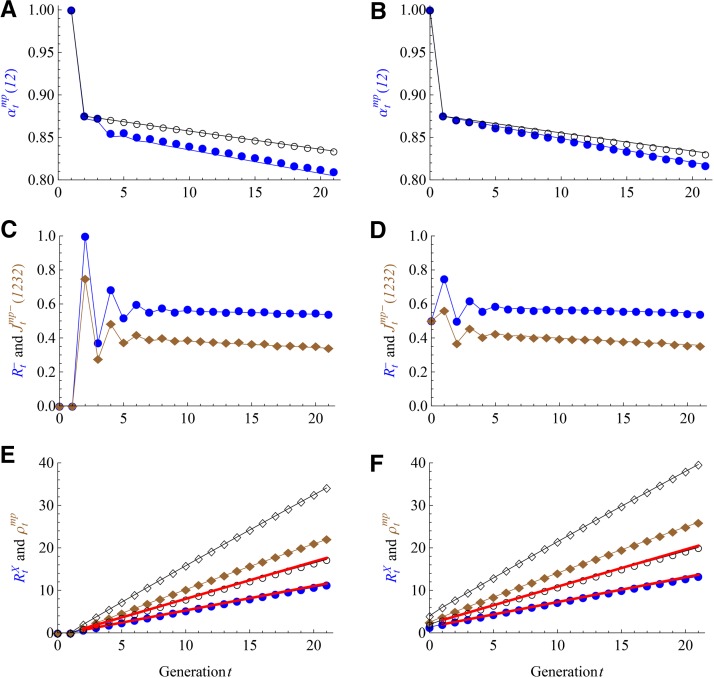
Results of the AIL (left panels) and the HS (right panels) with *L* = 8 and *N* = 100. The random mating schemes *M_F_* = RM1-NE for the AIL and RM1-E for the HS, and *M_I_* = RM1-E for both populations. The symbols and lines are the same as those in Figure 3. The theoretical predictions refer to Table 2 for the AIL and Table 3 for the DO. The additional red lines denote the map expansions under the large size assumption, given by equations (12a, 12b) for the AIL and equations (13a, 13b) for the HS.

The left panels of Figure 4 show for the AIL that the theoretical predictions fit very well with the forward simulation results, where ${ℳ}_{F}=$ RM1-NE, ${ℳ}_{I}=$ RM1-E, L=8, and N=100. Within U=20 intercross generations, the non-IBD probability $\alpha_{t}^{mp}\left(12\right)$ decreases slowly with generation *t*, the differential map expansion $R_{t}^{-}$ remains almost constant after a few generations of oscillations, and the map expansions in equations (12a, 12b), shown as thick red lines in Figure 4, are very good approximations.

### HS and DO

The HS and the DO differ from the AIL only in the genetic compositions of the founder population. The *N* progenitors of the DO at t=0 were sampled independently from pre-CC lines at a variety of different generations. Each pre-CC line is produced by the RIL by sibling mating from L=8 randomly permuted founder strains. Let $q_{k}$ denote the proportion of the pre-CC progenitors that were in generation *k*. Thus, for a random progenitor, it holds $\alpha_{0}^{mp}\left(12\right)=\sum_{k}q_{k}\alpha_{k}^{mp}\left(12|\text{pre}‐\text{CC}\right)$ and $R_{0}^{O}=\sum_{k}q_{k}R_{k}^{O}\left(\text{pre}‐\text{CC}\right)$, where $\alpha_{k}^{mp}\left(12|\text{pre}‐\text{CC}\right)$ and $R_{k}^{O}\left(\text{pre}‐\text{CC}\right)$ for O=m,p can be obtained from Table 1. Because the founder stains are exchangeable, we obtain $\beta_{0}^{mm}\left(12\right)=\beta_{0}^{mp}\left(12\right)=\beta_{0}^{pp}\left(12\right)=1-1/L$, $\alpha_{0}^{mmp}\left(123\right)=\alpha_{0}^{mpp}\left(123\right)=\alpha_{0}^{mp}\left(12\right)\left(1-2/L\right)$, and $\beta_{0}^{mmm}\left(123\right)=\beta_{0}^{mmp}\left(123\right)=\beta_{0}^{mpp}\left(123\right)=\beta_{0}^{ppp}\left(123\right)=\left(1-1/L\right)\left(1-2/L\right)$, and because recombination crossovers are independent among different pre-CC lines, the between-individual expected junction densities at t=0 are given by $K_{0}^{mm}\left(1122\right)=K_{0}^{mp}\left(1122\right)=K_{0}^{pp}\left(1122\right)=0$, $K_{0}^{mm}\left(1232\right)=K_{0}^{mp}\left(1232\right)=R_{0}^{m}\left(1-2/L\right)$, and $K_{0}^{pp}\left(1232\right)=K_{0}^{pm}\left(1232\right)=R_{0}^{p}\left(1-2/L\right)$, where 1−2/L refers to the probability that the third ancestral origin on haplotype bd is different from the two ancestral origins on haplotype ac where the ancestry transition occurs. The within-individual expected junction densities at t=0 are not required in the following derivations.

The $F_{1}$ population of size *N* is produced by random mating with equal sex ratio. Assuming that the population size *N* » 6, the coalescence probabilities at t=1 are approximated to be zero. According to the recurrence equations for the two- and three-gene non-IBD probabilities, the between-individual probabilities did not change and the within-individual non-IBD probabilities at t=1 equal to the corresponding between-individual probabilities. In addition, we have $R_{1}^{m}=R_{0}^{m}/2+R_{0}^{p}/2+\alpha_{0}^{mp}\left(12\right)$, $R_{1}^{p}=R_{0}^{m}$, $K_{1}^{mm}\left(1122\right)=K_{1}^{mp}\left(1122\right)=K_{1}^{pp}\left(1122\right)=0$, $K_{1}^{mm}\left(1232\right)=K_{1}^{mp}\left(1232\right)=\left[R_{0}^{m}/2+R_{0}^{p}/2+\alpha_{0}^{mp}\left(12\right)\right]\left(1-2/L\right)$, $K_{1}^{pp}\left(1232\right)=K_{1}^{pm}\left(1232\right)=R_{0}^{m}\left(1-2/L\right)$, according to the recurrence equations for the expected junction densities.

Similar to the intercross stage of the AIL, we obtain in Table 3 the results for X chromosomes in the DO in the last generation t=U+1 by substituting the initial conditions in the $F_{1}$ population into the theorems of Appendix A3. Table 3 also shows the results for autosomes, which are derived according to Zheng *et al.* (2014). Under the limit of an infinitely large population size (*N*→∞), we obtain from Table 3

$$R_{U+1}^{X}\approx R_{0}^{X}+\frac{2}{3}\alpha_{0}^{mp}\left(12\right)+\frac{2}{3}\left(1-\frac{1}{L}\right)U,$$ (13a)

$$R_{U+1}^{A}\approx R_{0}^{A}+\alpha_{0}^{AA}\left(12\right)+\left(1-\frac{1}{L}\right)U,$$ (13b)

showing that the two-thirds rule is valid under such an approximation since $\alpha_{0}^{mp}\left(12\right)=\alpha_{0}^{AA}\left(12\right)$ and $R_{0}^{X}=2R_{0}^{A}/3$ for progenitors drawn from the RIL (Table 1). The map expansion in equation (13b) for L=8 is the same as the one obtained by Broman (2012b).

The right panels of Figure 4 show for the HS that the theoretical predictions fit very well with the forward simulation results, where ${ℳ}_{F}={ℳ}_{I}=$ RM1-E, the N= 100 individuals in the $F_{0}$ population were sampled independently from CC funnels at the same generation t=3. The results are similar to those for the AIL with the same *L* shown in the left panels of Figure 4. For X chromosomes, the non-IBD probabilities in the DO are larger than those in the AIL, and thus in the DO the map expands at a higher rate than that for the AIL, see equations (12a, 13a).

### DSPR

The DSPR RILs were derived from two synthetic populations, each created independently by adding the multiparental AIL with an inbreeding stage by sibling mating (King *et al.* 2012). For example, we derive the analytic expressions of the map expansions in one synthetic population with *L* founder strains. We assume that $\beta_{U+1}^{mm}\left(12\right)=\beta_{U+1}^{mp}\left(12\right)$, which holds in a non-inbreeding population and approximately in a large population (*e.g.*, N≥100) with a large number of intercross generations (*e.g.*, U≥6). According to the map expansions in Table S3, we have

$$R_{U+V+1}^{X}\approx R_{U+1}^{X}+\alpha_{U+1}^{mp}\left(12\right)\left[4-\frac{30+14\sqrt{5}}{15}{\left(\lambda_{1}\right)}^{V}-\frac{30-14\sqrt{5}}{15}{\left(\lambda_{2}\right)}^{V}\right],$$ (14a)

$$R_{U+V+1}^{A}\approx R_{U+1}^{A}+\alpha_{U+1}^{AA}\left(12\right)\left[6-\frac{15+7\sqrt{5}}{5}{\left(\lambda_{1}\right)}^{V}-\frac{15-7\sqrt{5}}{5}{\left(\lambda_{2}\right)}^{V}\right],$$ (14b)

where $\lambda_{1}=\left(1+\sqrt{5}\right)/4$ and $\lambda_{2}=\left(1-\sqrt{5}\right)/4$, and $R_{U+1}^{X}$ and $\alpha_{U+1}^{mp}\left(12\right)$ are given in Table 1, Table 2, or Table 3 if the $F_{U+1}$ population is the last generation of the RIL, the AIL, or the DO, respectively.

**Figure 5:**
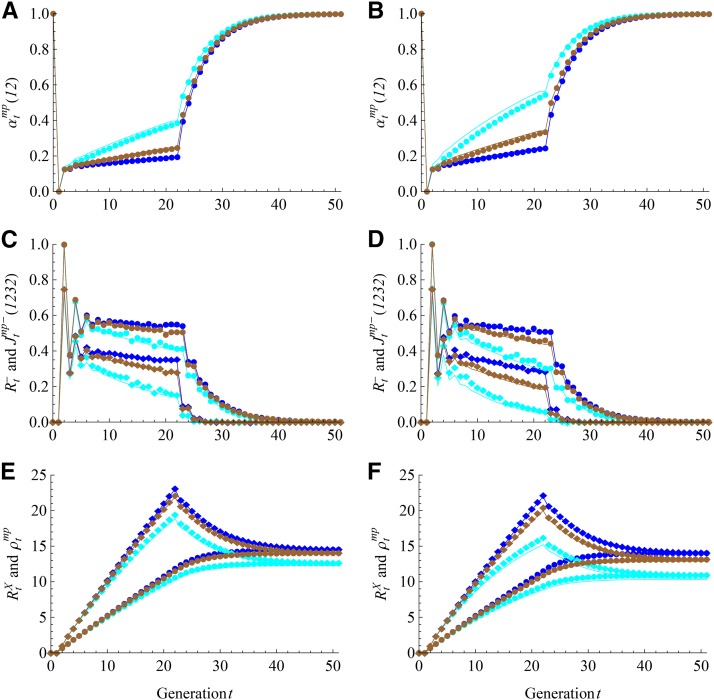
Results of the DSPR for X chromosomes with L=8 and N= 20 (cyan), 50 (brown), and 100 (blue). The random mating schemes *M_F_* = RM1-NE for all panels and *M_I_* = RM1-E (RM1-NE) for the left (right) panels. The lines denote the theoretical predictions under the large size assumption. The filled circles refer to $R_{t}^{-}$ in panels C and D, and $R_{t}^{X}$ in panels E and F; the filled diamonds refer to $J_{t}^{mp-}\left(1232\right)$ in (C) and (D) and $\rho_{t}^{mp}$ in (E) and (F).

We evaluate the large size assumption for various random mating schemes by simulation studies of the DSPR. Figure 5 shows the fitting of the theoretical predictions with the forward simulation results for the intercross size N= 20, 50, and 100, where the mating schemes ${ℳ}_{F}=$ RM1-NE and ${ℳ}_{I}=$ RM1-E (RM1-NE) for the left (right) panels. The theoretical predictions are obtained by combining the results for the AIL (Table 2) with those for the sibling-mating population (Table S3), assuming the large size (*N* » 6). The relative worse fitting for the differential densities $R_{t}^{-}$ and $J_{t}^{mp-}\left(1232\right)$ is probably attributable to the limited number (2 × 10^4^) of simulation replicates. The theoretical fitting becomes improved with increased size *N*, and it is very good for *N* = 100 within the range of *U* = 20 intercross generations. The fitting for RM1-E is better than RM1-NE because in the former case the two-gene coalescence probabilities are always equal to the three-gene probabilities (Table S2), independent of the size *N*. Figure S2 shows similar results for the random mating scheme RM2, except that the expected junction densities are slightly smaller. Figure S3 and Figure S4 show that the large size assumption is less sensitive for autosomes, and the fittings are very good even for *N* = 20.

## Discussion

We have extended our previous framework of modeling ancestral origin processes from autosomes to X chromosomes, and thus the same assumptions such as exchangeability of ancestral origins, Markov properties and random mating also apply (Zheng *et al.* 2014). The deviations from Markov properties result in larger variances in the IBD-tract length and the junction densities, which have been shown to be acceptable (Chapman and Thompson 2003; Martin and Hospital 2011). The random mating indicates that our approach does not apply to breeding populations with marker-assisted selections.

In contrast to the previous approaches (Haldane and Waddington 1931; Broman 2012a), the exchangeability assumption of ancestral origins greatly reduces model complexity, because the number of possible junction types does not depend on the number of founders for *L* ≥ 3 whereas the number of diplotype states increases very fast with *L*. The assumption affects the rate matrix of the Markov model, but not the expected junction densities where only changes of ancestral origins matter. The exchangeability is a good approximation for the AIL- or the multiparent advanced generation inter-cross (*i.e.*, MAGIC)-type populations with random mating, but it does not hold for the multiway RIL by sibling mating.

However, the exchangeability assumption is not critical for the application of our results to haplotype reconstructions from genotype data. The genomes of the individuals collected in the last generation have been well mixed by random chromosomal segregations over many generations. This is demonstrated in Figure 1A for the four-way RIL by sibling mating, where a female A and a male B was crossed, and a female C and a male D was crossed, and then a daughter from A × B and a son from C × D was crossed. The X chromosome of the founder D is lost in $F_{1}$. The genotype probabilities for AB and AC are different and given in the Table 2 of Broman (2012a), although the sum of the genotype probabilities for AB, AC, and BC is equal to $\alpha_{t}^{mp}\left(12\right)$ in Table 1. Figure 1B shows that the genotype probability for AB or AC becomes close to the average probability $\alpha_{t}^{mp}\left(12\right)/3$ as generation *t* increases. Furthermore, in the beginning generations when the asymmetry among ancestral origins is large, there are fewer number of recombination breakpoints, and thus more marker data per genome block are available to estimate ancestral origins. As a result, *a priori* equal weights of ancestral origins have little effects.

An HMM is under development for reconstructing ancestral origins for both autosomes and X chromosomes from marker data, using the present model and the previous one (Zheng *et al.* 2014) as the prior distribution. The previously implemented HMM methods, such as GAIN (Liu *et al.* 2010) and HAPPY (Mott *et al.* 2000), were developed for autosomes, and they do not account for the asymmetry between maternally and paternally derived X chromosomes.

The closed form expressions for non-IBD probabilities and various expected junction densities have been derived for stage-wise mapping populations. They provide the complete information for constructing the CTMC along two X chromosomes but also the guides for designing a new population in terms of X-linked QTL mapping resolutions. For advanced intercross populations such as the AIL, the HS, and the DO under the assumption of a large intercross size, the map expands linearly at a rate proportional to the inverse of the number *L* of inbred founders, which is robust to intercross mating schemes. For the RIL and the inbreeding stage of the DSPR, the map expansion slows down with increasing level of inbreeding. The overall junction density $\rho^{mp}$ for the DSPR decreases after one generation of the inbreeding stage by sibling mating, whereas for the RIL it reaches the maximum in the middle of inbreeding by sibling mating. These conclusions can also be applied to autosomes. Thus the most effective way of improving mapping resolutions is to increase the number *U* of intercross generations in a large population (*N* ≥ 5*U*, empirically).

## Appendix A

### Results for constant random mating populations

We introduce some matrix-vector notations to facilitate the derivations. Denote by A⊙B the element-by-element multiplication of the two matrices A and B, and by A⊘B the element-by-element division of the two matrices. Denote by $x_{i-j}^{t}=\left(x_{i}^{t},x_{i+1}^{t},\ldots ,x_{j}^{t}\right)$ the element-wise power where the subscripts of the natural numbers i≤j, and by default $x_{i-j}=\left(x_{i},x_{i+1},\;\ldots ,x_{j}\right)$. Let **1** be a vector with appropriate length and all the elements being 1. Let Λ(x) be the diagonal matrix with the diagonal elements being the vector x. Denote by [x, …,y] the matrix with row vectors x, …, y of equal length. Denote by superscript *T* the transpose of a vector or matrix.

The closed form expressions for the two- and three-gene non-IBD probabilities and the expected junction densities are derived for populations with constant size and random mating schemes. The coalescence probabilities are thus constant, and set $s_{t}^{m}=s^{m}$, $s_{t}^{p}=s^{p}$, $q_{t}^{m}=q^{m}$, and $q_{t}^{p}=q^{p}$. We first consider a finite population, number of males $N_{m}\geq 1$ and number of females $N_{f}\geq 3$, so that all the two- and three-gene non-IBD probabilities exist. Then consider an example of small population size, a sibling-mating population with one male and one female ($N_{f}=N_{m}=1$), where the non-IBD probabilities $\beta^{pp}\left(12\right)$, $\beta^{mmm}\left(123\right)$, $\beta^{mmp}\left(123\right)$, $\beta^{mpp}\left(123\right)$, and $\beta^{ppp}\left(123\right)$ do not exist. Lastly, we consider a large population under the limit that the size $N_{f}=N_{m}\gg 3$.

### A1 Finite population

**Definition A1.1.** The finite population refers to a population of constant number $N_{m}\geq 1$ of males and number $N_{f}\geq 3$ of females, maintained by random mating, and the initial population satisfies $\alpha_{0}^{mp}\left(12\right)=\beta_{0}^{mp}\left(12\right)$, $\alpha_{0}^{mmp}\left(123\right)=\beta_{0}^{mmp}\left(123\right)$, and $\alpha_{0}^{mpp}\left(123\right)=\beta_{0}^{mpp}\left(123\right)$.

**Proposition A1.2.** *Denote by* $\beta_{t}\left(12\right)={\left(\beta_{t}^{mm}\left(12\right),\beta_{t}^{mp}\left(12\right),\beta_{t}^{pp}\left(12\right)\right)}^{T}$ the two-gene non-IBD probability in a finite population. According to equations (4a–4c), it holds

$$\beta_{t}\left(12\right)=T_{12}\beta_{t-1}\left(12\right)$$ (A1.1)

*where*

$$T_{12}=\left(\begin{array}{ccc} \frac{1}{4}\left(1-s^{m}\right) & \frac{1}{2} & \frac{1}{4}\left(1-s^{m}\right)\\ \frac{1}{2} & \frac{1}{2} & 0\\ 1-s^{p} & 0 & 0 \end{array}\right).$$ (A1.2)

**Premise A1.3.** *The eigenvalues of* $T_{12}$ in a finite population, denoted by $\lambda_{k}$, k∈{1,2,3} in the decreasing order of their absolute values, are distinct with multiplicities 1, and none of them is 0, 1, or −1/2.

**Theorem A1.4.** *The two-gene non-IBD probability* $\beta_{t}\left(12\right)$ in a finite population is given by

$$\beta_{t}\left(12\right)=P\Lambda \left(\lambda_{1-3}^{t}\right){\left(C_{1-3}\right)}^{T},$$ (A1.3)

*where*

$$P=\left[2\lambda_{1-3}-1,1,\left(1-s^{p}\right)\left(2\lambda_{1-3}-1\right)⊘\lambda_{1-3}\right],$$ (A1.4)

$${\left(C_{1-3}\right)}^{T}=P^{-1}\beta_{0}\left(12\right).$$ (A1.5)

*Proof*. It holds

$$\beta_{t}^{mm}\left(12\right)=B_{1-3}{\left(\lambda_{1-3}^{t}\right)}^{T},$$ (A1.6a)

$$\beta_{t}^{mp}\left(12\right)=C_{1-3}{\left(\lambda_{1-3}^{t}\right)}^{T},$$ (A1.6b)

$$\beta_{t}^{pp}\left(12\right)=D_{1-3}{\left(\lambda_{1-3}^{t}\right)}^{T},$$ (A1.6c)

where the constant coefficients $B_{1-3}$, $C_{1-3}$, and $D_{1-3}$ are to be solved. Substituting $\beta_{t}^{mm}\left(12\right)$ and $\beta_{t}^{mp}\left(12\right)$ of equations (A1.6a, A1.6b) into the recurrence equation (4b), we obtain

$$B_{k}=\left(2\lambda_{k}-1\right)C_{k},k\in \left\{1,2,3\right\}.$$ (A1.7)

Substituting $\beta_{t}^{mm}\left(12\right)$ and $\beta_{t}^{pp}\left(12\right)$ of equations (A1.6a, A1.6c) into the recurrence equation (4c), we obtain

$$D_{k}=\left(1-s^{p}\right)\frac{B_{k}}{\lambda_{k}},k\in \left\{1,2,3\right\},$$ (A1.8)

Substituting $B_{k}$ and $D_{k}$ of equations (A1.7, A1.8) into equation (A1.6a–A1.6c), we obtain

$$\beta_{t}\left(12\right)=P\left[C_{1-3}{\left(\lambda_{1-3}^{t}\right)}^{T}\right],$$ (A1.9)

where P is given by in equation (A1.4), and the constant coefficient $C_{1-3}$ is determined by the initial condition $\beta_{0}\left(12\right)$ and it is given by equation (A1.5).

**Proposition A1.5.** *Denote by* $\beta_{t}\left(123\right)={\left(\beta_{t}^{mmm}\left(123\right),\beta_{t}^{mmp}\left(123\right),\beta_{t}^{mpp}\left(123\right),\beta_{t}^{ppp}\left(123\right)\right)}^{T}$ the three-gene non-IBD probability in a finite population. According to equations (5a–5d), it holds

$$\beta_{t}\left(123\right)=T_{123}\beta_{t-1}\left(123\right)$$ (A1.10)

*where*

$$T_{123}=\left(\begin{array}{cccc} \frac{1}{8}\left(1-s^{m}-2q^{m}\right) & \frac{3}{8}\left(1-s^{m}\right) & \frac{3}{8}\left(1-s^{m}\right) & \frac{1}{8}\left(1-s^{m}-2q^{m}\right)\\ \frac{1}{4}\left(1-s^{m}\right) & \frac{1}{2} & \frac{1}{4}\left(1-s^{m}\right) & 0\\ \frac{1}{2}\left(1-s^{p}\right) & \frac{1}{2}\left(1-s^{p}\right) & 0 & 0\\ 1-s^{p}-2q^{p} & 0 & 0 & 0 \end{array}\right).$$ (A1.11)

**Premise A1.6.** *The eigenvalues of* $T_{123}$ in a finite population, denoted by $\lambda_{k},k\in \left\{4,5,6,7\right\}$ in the decreasing order of their absolute values, are distinct with multiplicities 1, and none of them is 0, 1, −1/2, or $\lambda_{k},k\in \left\{1,2,3\right\}$.

**Theorem A1.7.** *The three-gene non-IBD probability* $\beta_{t}\left(123\right)$ in a finite population is given by

$$\beta_{t}\left(123\right)=Q\Lambda \left(\lambda_{4-7}^{t}\right){\left(C_{4-7}\right)}^{T},$$ (A1.12)

*where*

$$Q=\left[a_{4-7},1,\left(1-s^{p}\right)\left(a_{4-7}+1\right)⊘\left(2\lambda_{4-7}\right),\left(1-s^{p}-2q^{p}\right)a_{4-7}⊘\lambda_{4-7}\right],$$ (A1.13)

$${\left(C_{4-7}\right)}^{T}=Q^{-1}\beta_{0}\left(123\right).$$ (A1.14)

*and for* k∈{4,5,6,7}

$$a_{k}=\frac{8{\left(\lambda_{k}\right)}^{2}-4\lambda_{k}-\left(1-s^{m}\right)\left(1-s^{p}\right)}{\left(1-s^{m}\right)\left(1-s^{p}+2\lambda_{k}\right)}.$$ (A1.15)

*Proof*. It holds

$$\beta_{t}^{mmm}\left(123\right)=A_{4-7}{\left(\lambda_{4-7}^{t}\right)}^{T},$$ (A1.16a)

$$\beta_{t}^{mmp}\left(123\right)=C_{4-7}{\left(\lambda_{4-7}^{t}\right)}^{T},$$ (A1.16b)

$$\beta_{t}^{mpp}\left(123\right)=B_{4-7}{\left(\lambda_{4-7}^{t}\right)}^{T},$$ (A1.16c)

$$\beta_{t}^{ppp}\left(123\right)=D_{4-7}{\left(\lambda_{4-7}^{t}\right)}^{T},$$ (A1.16d)

where the constant coefficients $A_{4-7}$, $B_{4-7}$, $C_{4-7}$, and $D_{4-7}$ are to be solved. Substituting $\beta_{t}^{mmm}\left(123\right)$ and $\beta_{t}^{ppp}\left(123\right)$ of equations (A1.16a, A1.16d) into the recurrence equation (5d), we obtain

$$D_{k}=\left(1-s^{p}-2q^{p}\right)\frac{A_{k}}{\lambda_{k}},k\in \left\{4,5,6,7\right\}.$$ (A1.17)

Substituting $\beta_{t}^{mmm}\left(123\right)$, $\beta_{t}^{mmp}\left(123\right)$, and $\beta_{t}^{mpp}\left(123\right)$ of equations (A1.16a–A1.16c) into the recurrence equation (5c), we obtain

$$B_{k}=\frac{\left(1-s^{p}\right)\left(A_{k}+C_{k}\right)}{2\lambda_{k}},k\in \left\{4,5,6,7\right\}.$$ (A1.18)

Substituting $\beta_{t}^{mmm}\left(123\right)$, $\beta_{t}^{mmp}\left(123\right)$, and $\beta_{t}^{mpp}\left(123\right)$ of equations (A1.16a–A1.16c) into the recurrence equation (5b), and substituting $B_{k}$ of equation (A1.18), we obtain

$$A_{k}=a_{k}C_{k},k\in \left\{4,5,6,7\right\},$$ (A1.19)

where $a_{k}$ is given by equation (A1.15). Substituting $A_{k}$, $B_{k}$, and $D_{k}$ of equations (A1.17–A1.19) into equations (A1.16a–A1.16d), we obtain

$$\beta_{t}\left(123\right)=Q\left[C_{4-7}{\left(\lambda_{4-7}^{t}\right)}^{T}\right],$$ (A1.20)

where Q is given by equation (A1.13), and the constant coefficient $C_{4-7}$ is determined by the initial condition $\beta_{0}\left(123\right)$ and it is given by equation (A1.14).

**Theorem A1.8.** *The map expansions in a finite population are given by*

$$R_{t}^{X}=C_{8}-\frac{2}{3}\left[C_{1-3}⊘\left(1-\lambda_{1-3}\right)\right]{\left(\lambda_{1-3}^{t}\right)}^{T},$$ (A1.21)

$$R_{t}^{m}=C_{8}+C_{9}{\left(-\frac{1}{2}\right)}^{t}+C_{10-12}{\left(\lambda_{1-3}^{t}\right)}^{T},$$ (A1.22)

$$R_{t}^{p}=C_{8}-2C_{9}{\left(-\frac{1}{2}\right)}^{t}+C_{10-12}{\left(\lambda_{1-3}^{t-1}\right)}^{T},$$ (A1.23)

*where* $C_{1-3}$ is given by equation (A1.5), *and*

$$C_{8}=R_{0}^{X}+\frac{2}{3}\left[C_{1-3}⊘\left(1-\lambda_{1-3}\right)\right],$$ (A1.24)

$$C_{9}=R_{0}^{m}-C_{8}-C_{10-12}1^{T},$$ (A1.25)

$$C_{10-12}=-2\left(C_{1-3}⊙\lambda_{1-3}\right)⊘\left[\left(1-\lambda_{1-3}\right)⊙\left(2\lambda_{1-3}+1\right)\right].$$ (A1.26)

*Proof*. According to equation (7a), $R_{t}^{X}$ can be obtained from the accumulative summation of the non-IBD probability $\beta_{t}^{mp}$ of equation (A1.6b), and we have

$$R_{t}^{X}=R_{0}^{X}+\frac{2}{3}\sum_{k=1}^{3}C_{k}\frac{1-{\left(\lambda_{k}\right)}^{t}}{1-\lambda_{k}},$$ (A1.27)

which is equivalent to equation (A1.21) with the stationary map expansion $C_{8}$ being given by equation (A1.24). The eigenvalues for the transition matrix of the linear recurrence equations (4a–4c) and equations (6a, 6b) are 1, −1/2, $\lambda_{1-3}$, and thus the map expansions $R^{m}$ and $R^{p}$ can be expressed in the forms of equations (A1.22, A1.23), where the constant coefficients $C_{10-12}$ are determined by calculating $R_{t}^{X}=2R_{t}^{m}/3+R_{t}^{p}/3$ from equations (A1.22, A1.23) and comparing the result with equation (A1.21), and $C_{9}$ is determined by the initial condition $R_{0}^{m}$. □

**Theorem A1.9.** *Denote by* $K_{t}\left(1122\right)={\left(K_{t}^{mm}\left(1122\right),K_{t}^{mp}\left(1122\right),K_{t}^{pp}\left(1122\right)\right)}^{T}$ the expected density of junction type (1122) in a finite population, and it holds

$$\begin{array}{c} K_{t}\left(1122\right)=C_{8}+{\left(1,-1/2,-2\right)}^{T}C_{9}{\left(-\frac{1}{2}\right)}^{t}+W\Lambda \left(\lambda_{1-3}^{t}\right){\left(C_{18-20}\right)}^{T}\\ +P\Lambda \left(\lambda_{1-3}^{t}\right)\left[{\left(C_{15-17}\right)}^{T}+{\left(C_{18-20}\right)}^{T}t\right] \end{array}$$ (A1.28)

*where* $C_{8-12}$ are given by equations (A1.24–A1.26), P is given by equation (A1.4),

$$W=\left[2\lambda_{1-3},0,\left(s^{p}\Phi_{1-3}^{-1}+\left(1-s^{p}\right)\right)⊘\lambda_{1-3}\right],$$ (A1.29)

$${\left(C_{15-17}\right)}^{T}=P^{-1}\left[K_{0}\left(1122\right)-C_{8}-{\left(1,-1/2,-2\right)}^{T}C_{9}-W{\left(C_{18-20}\right)}^{T}\right],$$ (A1.30)

$$C_{18-20}=\Phi_{1-3}⊙C_{10-12},$$ (A1.31)

*and for* k∈{1,2,3}

$$\Phi_{k}=\frac{s^{m}\left(\lambda_{k}+1\right)+\left(1-s^{m}\right)s^{p}}{\left(6-2s^{m}\right)\lambda_{k}^{2}+\left(6-2s^{m}-4s^{p}+4s^{m}s^{p}\right)\lambda_{k}-3\left(1-s^{m}\right)\left(1-s^{p}\right)}.$$ (A1.32)

*Proof*. The eigenvalues for the transition matrix of the linear recurrence equations (4a–4c), equations (6a, 6b), and equations (9a–9c) are 1, −1/2, and duplicated $\lambda_{1-3}$. It holds

$$K_{t}^{mm}\left(1122\right)=B_{13}+B_{14}{\left(-\frac{1}{2}\right)}^{t}+B_{15-17}{\left(\lambda_{1-3}^{t}\right)}^{T}+B_{18-20}t{\left(\lambda_{1-3}^{t}\right)}^{T},$$ (A1.33a)

$$K_{t}^{mp}\left(1122\right)=C_{13}+C_{14}{\left(-\frac{1}{2}\right)}^{t}+C_{15-17}{\left(\lambda_{1-3}^{t}\right)}^{T}+C_{18-20}t{\left(\lambda_{1-3}^{t}\right)}^{T},$$ (A1.33b)

$$K_{t}^{pp}\left(1122\right)=D_{13}+D_{14}{\left(-\frac{1}{2}\right)}^{t}+D_{15-17}{\left(\lambda_{1-3}^{t}\right)}^{T}+D_{18-20}t{\left(\lambda_{1-3}^{t}\right)}^{T},$$ (A1.33c)

where the constant coefficients $B_{13-20}$, $C_{13-20}$, and $D_{13-20}$ are to be solved. Substituting $K_{t}^{mm}\left(1122\right)$ and $K_{t}^{mp}\left(1122\right)$ of equations (A1.33a, A1.33b) into the recurrence equation (9b), we obtain

$$\begin{array}{c} B_{13}=C_{13},\\ B_{14}=-2C_{14},\\ B_{14+k}=\left(2\lambda_{k}-1\right)C_{14+k}+2\lambda_{k}C_{17+k},k\in \left\{1,2,3\right\},\\ B_{17+k}=\left(2\lambda_{k}-1\right)C_{17+k},k\in \left\{1,2,3\right\}. \end{array}$$ (A1.34)

Substituting $K_{t}^{mm}\left(1122\right)$ and $K_{t}^{pp}\left(1122\right)$ of equations (A1.33a, A1.33c) and $R_{t}^{m}$ of equation (A1.22) into the recurrence equation (9c), and substituting $B_{13-20}$ of equation (A1.34), we obtain

$$\begin{array}{c} D_{13}=C_{13}+s^{p}\left(C_{8}-C_{13}\right),\\ D_{14}=-2s^{p}C_{9}+4\left(1-s^{p}\right)C_{14},\\ D_{14+k}=\left(1-s^{p}\right)\frac{2\lambda_{k}-1}{\lambda_{k}}C_{14+k}+s^{p}\frac{C_{9+k}}{\lambda_{k}}+\left(1-s^{p}\right)\frac{C_{17+k}}{\lambda_{k}},k\in \left\{1,2,3\right\},\\ D_{17+k}=\left(1-s^{p}\right)\frac{2\lambda_{k}-1}{\lambda_{k}}C_{17+k},k\in \left\{1,2,3\right\}, \end{array}$$ (A1.35)

Substituting $K_{t}^{mp}\left(1122\right)$, $K_{t}^{mm}\left(1122\right)$ and $K_{t}^{pp}\left(1122\right)$ of equations (A1.33a–A1.33c) and $R_{t}^{m}$ and $R_{t}^{p}$ of equations (A1.22, A1.23) into the recurrence equation (9a), and substituting $B_{13-20}$ and $D_{13-20}$ of equations (A1.34, A1.35), we obtain $C_{18-20}$ in equation (A1.31) and

$$C_{13}=C_{8},$$ (A1.36a)

$$C_{14}=-\frac{1}{2}C_{9}.$$ (A1.36b)

The constant coefficient $C_{15-17}$ is determined by the initial condition $K_{0}\left(1122\right)$.

**Definition A1.10.** Denote by

$$K_{t}^{mp+}\left(1232\right)=\frac{K_{t}^{mp}\left(1232\right)+K_{t}^{pm}\left(1232\right)}{2},$$ (A1.37)

$$K_{t}^{mp-}\left(1232\right)=\frac{K_{t}^{mp}\left(1232\right)-K_{t}^{pm}\left(1232\right)}{2},$$ (A1.38)

and thus

$$K_{t}^{mp}\left(1232\right)=K_{t}^{mp+}\left(1232\right)+K_{t}^{mp-}\left(1232\right),$$ (A1.39)

$$K_{t}^{pm}\left(1232\right)=K_{t}^{mp+}\left(1232\right)-K_{t}^{mp-}\left(1232\right).$$ (A1.40)

**Proposition A1.11.** *According to Definition A1.10*, *the recurrence equations (10a–10d) in a finite population are transformed into*

$$\begin{array}{c} K_{t}^{mm}\left(1232\right)=\left(1-s^{m}\right)\frac{1}{4}K_{t-1}^{mm}\left(1232\right)+\frac{1}{2}K_{t-1}^{mp+}\left(1232\right)+\left(1-s^{m}\right)\frac{1}{4}K_{t-1}^{pp}\left(1232\right)\\ +\left(1-s^{m}\right)\frac{1}{2}\left[\beta_{t-1}^{mmp}\left(123\right)+\beta_{t-1}^{mpp}\left(123\right)\right], \end{array}$$ (A1.41a)

$$K_{t}^{mp+}\left(1232\right)=\frac{1}{2}K_{t-1}^{mm}\left(1232\right)+\frac{1}{2}K_{t-1}^{mp+}\left(1232\right)+\frac{1}{2}\beta_{t-1}^{mmp}\left(123\right),$$ (A1.41b)

$$K_{t}^{pp}\left(1232\right)=\left(1-s^{p}\right)K_{t-1}^{mm}\left(1232\right),$$ (A1.41c)

$$K_{t}^{mp-}\left(1232\right)=-\frac{1}{2}K_{t-1}^{mp-}\left(1232\right)+\frac{1}{2}\beta_{t-1}^{mmp}\left(123\right).$$ (A1.41d)

**Theorem A1.12.** *Denote by* $K_{t}\left(1232\right)={\left(K_{t}^{mm}\left(1232\right),K_{t}^{mp+}\left(1232\right),K_{t}^{pp}\left(1232\right)\right)}^{T}$ the expected density of junction type (1232) in a finite population, and it holds

$$K_{t}\left(1232\right)=P\Lambda \left(\lambda_{1-3}^{t}\right){\left(C_{21-23}\right)}^{T}+W\Lambda \left(\lambda_{4-7}^{t}\right){\left(C_{24-27}\right)}^{T},$$ (A1.42)

*where* P is given by equation (A1.4),

$$W=\left[-\Phi_{4-7}^{-1}+\left(2\lambda_{4-7}-1\right),1,\left(1-s^{p}\right)\left(-\Phi_{4-7}^{-1}+\left(2\lambda_{4-7}-1\right)\right)⊘\lambda_{4-7}\right],$$ (A1.43)

$${\left(C_{21-23}\right)}^{T}=P^{-1}\left[K_{0}\left(1232\right)-W{\left(C_{24-27}\right)}^{T}\right],$$ (A1.44)

$$C_{24-27}=\Phi_{4-7}⊙C_{4-7},$$ (A1.45)

*and for* k∈{4,5,6,7}

$$\Phi_{k}=\frac{\frac{1}{2}{\left(\lambda_{k}\right)}^{2}+\frac{1}{8}\left[\left(1-s^{p}\right)\left(a_{k}+1\right)/\lambda_{k}+1\right]\left(1-s^{m}\right)\lambda_{k}-\frac{1}{8}\left(1-s^{m}\right)\left(1-s^{p}\right)}{f_{12}\left(\lambda_{k}\right)},$$ (A1.46)

*where* $f_{12}\left(x\right)$ is the characteristic polynomial of the transition matrix $T_{12}$ of equation (A1.2).

*Proof*. The eigenvalues for the transition matrix of the linear recurrence equations (5a–5d) and equations (A1.41a–A1.41c) are $\lambda_{k}$, 1≤k≤7. It holds

$$K_{t}^{mm}\left(1232\right)=B_{21-27}{\left(\lambda_{1-7}^{t}\right)}^{T},$$ (A1.47a)

$$K_{t}^{mp+}\left(1232\right)=C_{21-27}{\left(\lambda_{1-7}^{t}\right)}^{T},$$ (A1.47b)

$$K_{t}^{pp}\left(1232\right)=D_{21-27}{\left(\lambda_{1-7}^{t}\right)}^{T},$$ (A1.47c)

where the constant coefficients $B_{21-27}$, $C_{21-27}$, and $D_{21-27}$ are to be solved. Substituting $K_{t}^{mm}\left(1232\right)$ and $K_{t}^{pp}\left(1232\right)$ of equations (A1.47a, A1.47c) into the recurrence equation (A1.41c), we obtain

$$D_{20+k}=\left(1-s^{p}\right)\frac{B_{20+k}}{\lambda_{k}},1\leq k\leq 7,$$ (A1.48)

Substituting $K_{t}^{mp+}\left(1232\right)$ and $K_{t}^{mm}\left(1232\right)$ of equations (A1.47a, A1.47b) and $\beta_{t}^{mmp}$ of equation (A1.16b) into the recurrence equation (A1.41b), we obtain

$$B_{20+k}=\{\begin{array}{ll} \left(2\lambda_{k}-1\right)C_{20+k} & k\in \left\{1,2,3\right\},\\ -C_{k}+\left(2\lambda_{k}-1\right)C_{20+k} & k\in \left\{4,5,6,7\right\}. \end{array}$$ (A1.49)

The theorem follows by substituting $K_{t}^{mm}\left(1232\right)$, $K_{t}^{mp+}\left(1232\right)$ and $K_{t}^{pp}\left(1232\right)$ of equations (A1.47a–A1.47c) and $\beta_{t}^{mmp}$ and $\beta_{t}^{mpp}$ of equations (A1.16b, A1.16c) into the recurrence equation (A1.41a), and substituting $B_{21-27}$ and $D_{21-27}$ of equations (A1.48, A1.49), where the constant coefficient $C_{21-23}$ in equation (A1.44) is determined by the initial condition $K_{0}\left(1232\right)$. □

**Theorem A1.13.** *The expected density* $K_{t}^{mp-}\left(1232\right)$ is given by

$$K_{t}^{mp-}\left(1232\right)=A_{28}{\left(-\frac{1}{2}\right)}^{t}+A_{24-27}{\left(\lambda_{4-7}^{t}\right)}^{T},$$ (A1.50)

*where*

$$A_{24-27}=C_{4-7}⊘\left(2\lambda_{4-7}+1\right),$$ (A1.51)

$$A_{28}=K_{0}^{mp-}\left(1232\right)-A_{24-27}1^{T}.$$ (A1.52)

*Proof*. The eigenvalues of the transition matrix of the linear recurrence equations (5a–5d) and equation (A1.41d): −1/2, $\lambda_{k}$, k∈{4,5,6,7}. Thus equation (A1.50) holds, where $A_{24-27}$ is determined by putting equations (A1.50, A1.16b) into equation (A1.41d), and the constant coefficient $A_{28}$ is determined by the initial condition $K_{0}^{mp-}\left(1232\right)$. □

**Corollary A1.14.** *The overall expected junction density* $\rho_{t}^{mp}$ of a female in a finite population is given by

$$\rho_{t}^{mp}=C_{8}-\frac{1}{2}C_{9}{\left(-\frac{1}{2}\right)}^{t}+\left[C_{10-12}⊙\left(1+\lambda_{1-3}^{-1}\right)-C_{15-17}-C_{18-20}t\right]{\left(\lambda_{1-3}^{t}\right)}^{T},$$ (A1.53)

*where* $C_{8-12}$ are given by equations (A1.24–A1.26), and $C_{15-20}$ by equations (A1.30, A1.31).

*Proof*. The corollary follows by substituting $R_{t}^{m}$ and $R_{t}^{p}$ of equations (A1.22, A1.23) and $K_{t}^{mp}\left(1122\right)$ of equation (A1.33b) into equation (3).

### A2 Sibling-mating population

**Definition A2.1.** The sibling-mating population refers to a population of constant size 2, one male and one female, maintained by sibling mating, and the initial population satisfies $\alpha_{0}^{mp}\left(12\right)=\beta_{0}^{mp}\left(12\right)$. In such a population, the coalescence probability $s^{m}=1$, and the coalescence probabilities $s^{p}$, $q^{m}$, $q^{p}$ are set to zero.

**Definition A2.2.** In a sibling-mating population, the non-IBD probabilities $\beta_{t}^{pp}\left(12\right)$, $\beta_{t}^{mmm}\left(123\right)$, $\beta_{t}^{mmp}\left(123\right)$, $\beta_{t}^{mpp}\left(123\right)$, and $\beta_{t}^{ppp}\left(123\right)$ are set to zero since they do not exist. Similarly the expected junction densities $K^{pp}\left(1122\right)$ and $K^{pp}\left(1232\right)$ are set to zero.

**Proposition A2.3.** *Denote by* $\beta_{t}\left(12\right)=(\beta_{t}^{mm}\left(12\right),\beta_{t}^{mp}\left(12\right)$ the two-gene non-IBD probability in a sibling-mating population. According to equations (4a, 4b), it holds

$$T_{12}=\left(\begin{array}{cc} 0 & \frac{1}{2}\\ \frac{1}{2} & \frac{1}{2} \end{array}\right),$$ (A2.1)

*and its eigenvalues are given by*

$$\lambda_{1}=\frac{1+\sqrt{5}}{4},\lambda_{2}=\frac{1-\sqrt{5}}{4}.$$ (A2.2)

**Definition A2.4.** In a sibling-mating population, the conjugate is obtained by replacing $\sqrt{5}$ with $-\sqrt{5}$ from the terms involving $\lambda_{1}$.

**Theorem A2.5.** *The two-gene non-IBD probability* $\beta_{t}\left(12\right)$ in a sibling-mating population is given by

$$\beta_{t}^{mm}\left(12\right)=\left[\frac{5-\sqrt{5}}{10}\beta_{0}^{mm}\left(12\right)+\frac{\sqrt{5}}{5}\beta_{0}^{mp}\left(12\right)\right]{\left(\lambda_{1}\right)}^{t}+conjugate$$ (A2.3)

$$\beta_{t}^{mp}\left(12\right)=\left[\frac{\sqrt{5}}{5}\beta_{0}^{mm}\left(12\right)+\frac{5+\sqrt{5}}{10}\beta_{0}^{mp}\left(12\right)\right]{\left(\lambda_{1}\right)}^{t}+conjugate$$ (A2.4)

*Proof*. Similar to Theorem A1.4, it holds

$$\beta_{t}\left(12\right)=P\Lambda \left(\lambda_{1-2}^{t}\right){\left(C_{1-2}\right)}^{T},$$ (A2.5)

where

$$P=\left[2\lambda_{1-2}-1,1\right],$$ (A2.6)

$${\left(C_{1-2}\right)}^{T}=P^{-1}\beta_{0}\left(12\right).$$ (A2.7)

The theorem follows by substituting P an $C_{1-2}$ into equation (A2.5). □

**Theorem A2.6.** *The three-gene non-IBD probability in a sibling-mating population is given by*

$$\alpha_{t}^{mmp}\left(123\right)=\alpha_{0}^{mmp}\left(123\right){\left(\frac{1}{2}\right)}^{t}.$$ (A2.8)

*Proof*. According to the recurrence equations (5b, 5e),

$$\alpha_{t}^{mmp}\left(123\right)=\frac{1}{2}\alpha_{t-1}^{mmp}\left(123\right).$$ (A2.9)

Thus it holds

$$\alpha_{t}^{mmp}\left(123\right)=C_{4}{\left(\lambda_{4}\right)}^{t},$$ (A2.10)

where

$$\lambda_{4}=1/2,$$ (A2.11)

$$C_{4}=\alpha_{0}^{mmp}\left(123\right).$$ (A2.12)

**Theorem A2.7.** *The map expansions in a sibling-mating population are given by*

$$R_{t}^{X}=C_{8}-\left[\frac{10+6\sqrt{5}}{15}\beta_{0}^{mm}\left(12\right)+\frac{20+8\sqrt{5}}{15}\beta_{0}^{mp}\left(12\right)\right]{\left(\lambda_{1}\right)}^{t}+conjugate,$$ (A2.13)

$$R_{t}^{m}=C_{8}+C_{9}{\left(-\frac{1}{2}\right)}^{t}-\left[\frac{5+\sqrt{5}}{5}\beta_{0}^{mm}\left(12\right)+\frac{5+3\sqrt{5}}{5}\beta_{0}^{mp}\left(12\right)\right]{\left(\lambda_{1}\right)}^{t}+conjugate$$ (A2.14)

$$R_{t}^{p}=C_{8}-2C_{9}{\left(-\frac{1}{2}\right)}^{t}-\left[\frac{4\sqrt{5}}{5}\beta_{0}^{mm}\left(12\right)+\frac{10+2\sqrt{5}}{5}\beta_{0}^{mp}\left(12\right)\right]{\left(\lambda_{1}\right)}^{t}+conjugate$$ (A2.15)

*where* $C_{8}$ and $C_{9}$ are given by

$$C_{8}=\frac{1}{3}\left(2R_{0}^{m}+R_{0}^{p}\right)+\frac{4}{3}\left(\beta_{0}^{mm}\left(12\right)+2\beta_{0}^{mp}\left(12\right)\right),$$ (A2.16)

$$C_{9}=\frac{1}{3}\left(R_{0}^{m}-R_{0}^{p}\right)+\frac{2}{3}\left(\beta_{0}^{mm}\left(12\right)-\beta_{0}^{mp}\left(12\right)\right),$$ (A2.17)

*Proof*. Similar to Theorem A1.8, the map expansions in a sibling-mating population are given by

$$R_{t}^{X}=C_{8}-\frac{2}{3}\left[C_{1-2}⊘\left(1-\lambda_{1-2}\right)\right]{\left(\lambda_{1-2}^{t}\right)}^{T},$$ (A2.18)

$$R_{t}^{m}=C_{8}+C_{9}{\left(-\frac{1}{2}\right)}^{t}+C_{10-11}{\left(\lambda_{1-2}^{t}\right)}^{T},$$ (A2.19)

$$R_{t}^{p}=C_{8}-2C_{9}{\left(-\frac{1}{2}\right)}^{t}+C_{10-11}{\left(\lambda_{1-2}^{t-1}\right)}^{T},$$ (A2.20)

where

$$C_{8}=R_{0}^{X}+\frac{2}{3}\left[C_{1-2}⊘\left(1-\lambda_{1-2}\right)\right],$$ (A2.21)

$$C_{9}=R_{0}^{m}-C_{8}-C_{10-11}1^{T},$$ (A2.22)

$$C_{10-11}=-2\left(C_{1-2}⊙\lambda_{1-2}\right)⊘\left[\left(1-\lambda_{1-2}\right)⊙\left(2\lambda_{1-2}+1\right)\right].$$ (A2.23)

The theorem follows by substituting $\lambda_{1-2}$ of equation (A2.2) and $C_{1-2}$ of equation (A2.7) into the aforementioned equations.

**Theorem A2.8.** *The expected density* $K_{t}\left(1122\right)={\left(K_{t}^{mm}\left(1122\right),K_{t}^{mp}\left(1122\right)\right)}^{T}$ in a sibling-mating population is given by

$$\begin{array}{c} K_{t}^{mm}\left(1122\right)=C_{8}+C_{9}{\left(-\frac{1}{2}\right)}^{t}\\ +\left[-\frac{5+\sqrt{5}}{5}\beta_{0}^{mm}\left(12\right)-\frac{5+4\sqrt{5}}{5}\beta_{0}^{mp}\left(12\right)\right]{\left(\lambda_{1}\right)}^{t}\\ +\left[\frac{5-\sqrt{5}}{10}K_{0}^{mm}\left(1122\right)+\frac{\sqrt{5}}{5}K_{0}^{mp}\left(1122\right)-\frac{1}{2}R_{0}^{m}-\frac{\sqrt{5}}{10}R_{0}^{p}\right]{\left(\lambda_{1}\right)}^{t}\\ +\left[-\frac{5-\sqrt{5}}{10}\beta_{0}^{mm}\left(12\right)-\frac{\sqrt{5}}{5}\beta_{0}^{mp}\left(12\right)\right]t{\left(\lambda_{1}\right)}^{t}+conjugate \end{array}$$ (A2.24)

$$\begin{array}{c} K_{t}^{mp}\left(1122\right)=C_{8}-\frac{1}{2}C_{9}{\left(-\frac{1}{2}\right)}^{t}\\ +\left[-\frac{5+3\sqrt{5}}{10}\beta_{0}^{mm}\left(12\right)-\frac{3+\sqrt{5}}{2}\beta_{0}^{mp}\left(12\right)\right]{\left(\lambda_{1}\right)}^{t}\\ +\left[\frac{\sqrt{5}}{5}K_{0}^{mm}\left(1122\right)+\frac{5+\sqrt{5}}{10}K_{0}^{mp}\left(1122\right)-\frac{1+\sqrt{5}}{4}R_{0}^{m}-\frac{5+\sqrt{5}}{20}R_{0}^{p}\right]{\left(\lambda_{1}\right)}^{t}\\ +\left[-\frac{\sqrt{5}}{5}\beta_{0}^{mm}\left(12\right)-\frac{5+\sqrt{5}}{10}\beta_{0}^{mp}\left(12\right)\right]t{\left(\lambda_{1}\right)}^{t}+conjugate \end{array}$$ (A2.25)

*where* $C_{8}$ and $C_{9}$ are given by equations (A2.16, A2.17), respectively.

*Proof*. Similar to Theorem A1.9, the expected density $K_{t}\left(1122\right)$ in a sibling-mating population is given by

$$\begin{array}{c} K_{t}\left(1122\right)=C_{8}+{\left(1,-1/2\right)}^{T}C_{9}{\left(-\frac{1}{2}\right)}^{t}+W\Lambda \left(\lambda_{1-2}^{t}\right){\left(C_{18-19}\right)}^{T}\\ +P\Lambda \left(\lambda_{1-2}^{t}\right)\left[{\left(C_{15-16}\right)}^{T}+{\left(C_{18-19}\right)}^{T}t\right] \end{array}$$ (A2.26)

where $C_{8}$ and $C_{9}$ are given by equations (A2.16, A2.17), respectively, and P is given by equation (A2.6), and

$$W=\left[2\lambda_{1-2},0\right],$$ (A2.27)

$${\left(C_{15-16}\right)}^{T}=P^{-1}\left[K_{0}\left(1122\right)-C_{8}-{\left(1,-1/2\right)}^{T}C_{9}-W{\left(C_{18-19}\right)}^{T}\right],$$ (A2.28)

$$C_{18-19}=\Phi_{1-2}⊙C_{10-11},$$ (A2.29)

and for k∈{1,2}

$$\Phi_{k}=\frac{1}{4\lambda_{k}}.$$ (A2.30)

The theorem follows by substituting $\lambda_{1-2}$ of equation (A2.2) and $C_{10-11}$ of equation (A2.23).

**Theorem A2.9.** *The expected densities of type (1232) in a sibling-mating population are given by*

$$\begin{array}{c} K_{t}^{mm}\left(1232\right)=\left[\frac{5-\sqrt{5}}{10}K_{0}^{mm}\left(1232\right)+\frac{\sqrt{5}}{5}K_{0}^{mp+}\left(1232\right)+\frac{5+\sqrt{5}}{10}\alpha_{0}^{mmp}\left(123\right)\right]{\left(\lambda_{1}\right)}^{t}\\ -\alpha_{0}^{mmp}\left(123\right){\left(\frac{1}{2}\right)}^{t}+conjutate \end{array}$$ (A2.31)

$$\begin{array}{c} K_{t}^{mp+}\left(1232\right)=\left[\frac{\sqrt{5}}{5}K_{0}^{mm}\left(1232\right)+\frac{5+\sqrt{5}}{10}K_{0}^{mp+}\left(1232\right)+\frac{5+3\sqrt{5}}{10}\alpha_{0}^{mmp}\left(123\right)\right]{\left(\lambda_{1}\right)}^{t}\\ -\alpha_{0}^{mmp}\left(123\right){\left(\frac{1}{2}\right)}^{t}+conjutate, \end{array}$$ (A2.32)

$$K_{t}^{mp-}\left(1232\right)=\left[K_{0}^{mp-}\left(1232\right)-\frac{1}{2}\alpha_{0}^{mmp}\left(123\right)\right]{\left(-\frac{1}{2}\right)}^{t}+\frac{1}{2}\alpha_{0}^{mmp}\left(123\right){\left(\frac{1}{2}\right)}^{t}$$ (A2.33)

*Proof*. Denote by $K_{t}\left(1232\right)={\left(K_{t}^{mm}\left(1232\right),K_{t}^{mp+}\left(1232\right)\right)}^{T}$. Similar to Theorem A1.12, the expected density $K_{t}\left(1232\right)$ in a sibling-mating population is given by

$$K_{t}\left(1232\right)=P\Lambda \left(\lambda_{1-2}^{t}\right){\left(C_{21-22}\right)}^{T}+W\Lambda \left(\lambda_{4-4}^{t}\right){\left(C_{24-24}\right)}^{T},$$ (A2.34)

where P is given by equation (A2.6), and

$$W=\left[-\Phi_{4-4}^{-1}+\left(2\lambda_{4-4}-1\right),1\right],$$ (A2.35)

$${\left(C_{21-22}\right)}^{T}=P^{-1}\left[K_{0}\left(1232\right)-W{\left(C_{24-24}\right)}^{T}\right],$$ (A2.36)

$$C_{24-24}=\Phi_{4-4}⊙C_{4-4},$$ (A2.37)

and for k=4

$$\Phi_{k}=\frac{\frac{1}{2}\lambda_{k}}{f_{12}\left(\lambda_{k}\right)},$$ (A2.38)

where $f_{12}\left(x\right)$ is the characteristic polynomial of the transition matrix $T_{12}$ of equation (A2.1). The equations (A2.31, A2.32) follow by substituting $\lambda_{1-2}$ of equation (A2.2), $\lambda_{4}$ of equation (A2.11), and $C_{4}$ of equation (A2.12).

Similar to Theorem A1.13, the expected density $K_{t}^{mp-}\left(1232\right)$ in a sibling-mating population is given by

$$K_{t}^{mp-}\left(1232\right)=A_{28}{\left(-\frac{1}{2}\right)}^{t}+A_{24-24}{\left(\lambda_{4-4}^{t}\right)}^{T}$$ (A2.39)

where

$$A_{24-24}=C_{4-4}⊘\left(2\lambda_{4-4}+1\right),$$ (A2.40)

$$A_{28}=K_{0}^{mp-}\left(1232\right)-A_{24-24}.$$ (A2.41)

**Corollary A2.10.** *The overall expected junction density* $\rho_{t}^{mp}$ in a sibling-mating population is given by

$$\begin{array}{c} \rho_{t}^{mp}=C_{8}-\frac{1}{2}C_{9}{\left(-\frac{1}{2}\right)}^{t}\\ +\left[-\frac{5+7\sqrt{5}}{10}\beta_{0}^{mm}\left(12\right)-\frac{3+\sqrt{5}}{2}\beta_{0}^{mp}\left(12\right)\right]{\left(\lambda_{1}\right)}^{t}\\ +\left[-\frac{\sqrt{5}}{5}K_{0}^{mm}\left(1122\right)-\frac{5+\sqrt{5}}{10}K_{0}^{mp}\left(1122\right)+\frac{1+\sqrt{5}}{4}R_{0}^{m}+\frac{5+\sqrt{5}}{20}R_{0}^{p}\right]{\left(\lambda_{1}\right)}^{t}\\ +\left[\frac{\sqrt{5}}{5}\beta_{0}^{mm}\left(12\right)+\frac{5+\sqrt{5}}{10}\beta_{0}^{mp}\left(12\right)\right]t{\left(\lambda_{1}\right)}^{t}+conjutate, \end{array}$$ (A2.42)

*where* $C_{8}$ and $C_{9}$ are given by equations (A2.16, A2.17), respectively.

*Proof*. The corollary follows by substituting $R_{t}^{m}$ and $R_{t}^{p}$ of equations (A2.14, A2.15) and $K_{t}^{mp}\left(1122\right)$ of equation (A2.25) into equation (3).

### A3 Large population

**Definition A3.1.** The large population refers to a population with large and equal number $N_{m}=N_{f}\gg 3$ of males and females, maintained by random mating, and the initial population satisfies $\alpha_{0}^{mp}\left(12\right)=\beta_{0}^{mp}\left(12\right)$, $\alpha_{0}^{mmp}\left(123\right)=\beta_{0}^{mmp}\left(123\right)$, and $\alpha_{0}^{mpp}\left(123\right)=\beta_{0}^{mpp}\left(123\right)$. In such a large population, the coalescence probabilities are set to $s^{m}=s^{q}=q^{m}=q^{p}=s$.

**Proposition A3.2.** *Denote by* $\beta_{t}\left(12\right)=\left(\beta_{t}^{mm}\left(12\right),\beta_{t}^{mp}\left(12\right),\beta_{t}^{pp}\left(12\right)\right)$ the two-gene non-IBD probability in a large population, and the eigenvalues of the transition matrix $T_{12}$ in equation (A1.2) *are given by*

$$\lambda_{1}=1-\frac{s}{3},\lambda_{2}=-\frac{1}{2},\lambda_{3}=\frac{1}{4}.$$ (A3.1)

*where* $\lambda_{1}$ is approximated to the first order of s to keep it smaller than 1, and $\lambda_{2}$ and $\lambda_{3}$ are approximated to zero order of s.

**Theorem A3.3.** *The two-gene non-IBD probabilities in a large population are given by*

$$\beta_{t}\left(12\right)=P\Lambda \left(\lambda_{1-3}^{t}\right){\left(C_{1-3}\right)}^{T},$$ (A3.2)

*where*

$$P=\left[\begin{array}{ccc} 1 & -2 & -1/2\\ 1 & 1 & 1\\ 1 & 4 & -2 \end{array}\right],$$ (A3.3)

$${\left(C_{1-3}\right)}^{T}=\frac{1}{9}\left[\begin{array}{ccc} 4 & 4 & 1\\ -2 & 1 & 1\\ -2 & 4 & -2 \end{array}\right]\beta_{0}\left(12\right).$$ (A3.4)

(A3.5)

*Proof*. The theorem follows directly from Theorem A1.4, where

$$P=\left[2\lambda_{1-3}-1,1,\left(1-s\right)\left(2\lambda_{1-3}-1\right)⊘\lambda_{1-3}\right],$$ (A3.6)

$${\left(C_{1-3}\right)}^{T}=P^{-1}\beta_{0}\left(12\right).$$ (A3.7)

The matrix P is approximated to the zero order of *s*. □

**Proposition A3.4.** *Denote by* $\beta_{t}\left(123\right)={\left(\beta_{t}^{mmm}\left(123\right),\beta_{t}^{mmp}\left(123\right),\beta_{t}^{mpp}\left(123\right),\beta_{t}^{ppp}\left(123\right)\right)}^{T}$ the three-gene non-IBD probability in a large population, and the eigenvalues of the transition matrix $T_{123}$ of equation (A1.11) *are given by*

$$\lambda_{4}=1-s,\lambda_{5}=-\frac{1}{2},\lambda_{6}=\frac{1}{4},\lambda_{7}=-\frac{1}{8},$$ (A3.8)

*where* $\lambda_{4}$ is approximated to the first order of s to keep it smaller than 1, and $\lambda_{5-7}$ is approximated to zero order of s.

**Theorem A3.5.** *The three-gene non-IBD probability* $\beta_{t}\left(123\right)$ in a large population is given by

$$\beta_{t}\left(123\right)=Q\Lambda \left(\lambda_{4-7}^{t}\right){\left(C_{4-7}\right)}^{T},$$ (A3.9)

*where*

$$Q=\left[\begin{array}{cccc} 1 & -\frac{3}{s} & -1 & -\frac{1}{2}\\ 1 & 1 & 1 & 1\\ 1 & \frac{3}{s} & 0 & -2\\ 1 & \frac{6}{s} & -4 & 4 \end{array}\right],$$ (A3.10)

$${\left(C_{4-7}\right)}^{T}=Q^{-1}\beta_{0}\left(123\right).$$ (A3.11)

*Proof*. The theorem follows directly from Theorem A1.7, where

$$a_{k}=\frac{8{\left(\lambda_{k}\right)}^{2}-4\lambda_{k}-{\left(1-s\right)}^{2}}{\left(1-s\right)\left(1-s+2\lambda_{k}\right)},k\in \left\{4,5,6,7\right\},$$

$$Q=\left[a_{4-7},1,\left(1-s\right)\left(a_{4-7}+1\right)⊘\left(2\lambda_{4-7}\right),\left(1-3s\right)a_{4-7}⊘\lambda_{4-7}\right],$$

where the elements of Q are approximated to be the leading order of *s* and up to the zero order of *s*. □

**Theorem A3.6.** *The map expansions in a large population are given by*

$$R_{t}^{X}=R_{0}^{X}+\frac{2}{3}C_{1}\frac{\left[1-{\left(\lambda_{1}\right)}^{t}\right]}{1-\lambda_{1}}+\frac{4}{9}C_{2}\left[1-{\left(\lambda_{2}\right)}^{t}\right]+\frac{8}{9}C_{3}\left[1-{\left(\lambda_{3}\right)}^{t}\right],$$ (A3.12)

$$R_{t}^{m}=C_{8}+C_{10}\frac{1-{\left(\lambda_{1}\right)}^{t}}{1-\lambda_{1}}+\left(C_{9}+C_{11}t\right){\left(\lambda_{2}\right)}^{t}+C_{12}{\left(\lambda_{3}\right)}^{t},$$ (A3.13)

$$R_{t}^{p}=C_{8}+C_{10}\frac{1-{\left(\lambda_{1}\right)}^{t-1}}{1-\lambda_{1}}+\left[C_{9}+C_{11}\left(t-1\right)\right]{\left(\lambda_{2}\right)}^{t-1}+C_{12}{\left(\lambda_{3}\right)}^{t-1},$$ (A3.14)

*where* $C_{1-3}$ is given by equation (A3.4), *and*

$$C_{8}=R_{0}^{X}+\frac{2}{3\left(2\lambda_{1}+1\right)}C_{1}+\frac{4}{9}C_{2}+\frac{8}{9}C_{3},$$ (A3.15)

$$C_{9}=R_{0}^{m}-C_{8}-C_{12},$$ (A3.16)

$$C_{10}=\frac{2\lambda_{1}}{2\lambda_{1}+1}C_{1},C_{11}=-\frac{2}{3}C_{2},C_{12}=-\frac{4}{9}C_{3}.$$ (A3.17)

*Remark*. As s→0 given *t*, $\left[1-{\left(\lambda_{1}\right)}^{t}\right]/\left(1-\lambda_{1}\right)\to t$, that is, the map expansions are dominant by the linear term proportional to *t*; as t→∞ given *s*, the map expansions asymptotically go to the constant $C_{8}+C_{10}/\left(1-\lambda_{1}\right)$.

*Proof*. The proof is similar to Theorem A1.8, except that the eigenvalue $\lambda_{2}$ or −1/2 has multiplicity 2. □

**Theorem A3.7.** *The expected density* $K_{t}\left(1122\right)=\left(K_{t}^{mm}\left(1122\right),K_{t}^{mp}\left(1122\right),K_{t}^{pp}\left(1122\right)\right)$, *is given by*

$$K_{t}\left(1122\right)=C_{8}+C_{10}\frac{1-\lambda_{1}^{t}}{1-\lambda_{1}}-\left[1+\left(\frac{2}{9},\frac{8}{9},-\frac{4}{9}\right)s\right]C_{10}t\lambda_{1}^{t}+P\Lambda \left(\lambda_{1-3}^{t}\right){\left(C_{15-17}\right)}^{T},$$ (A3.18)

*where* $C_{8}$, $C_{10}$, and P are given by equation (A3.15), *equation* (A3.17), *and equation* (A3.3), *respectively*, *and*

$$C_{15-17}=P^{-1}\left[K_{0}\left(1122\right)-C_{8}\right].$$ (A3.19)

*Proof*. The eigenvalues for the transition matrix of the linear recurrence equations (4a–4c), equations (6a, 6b), and equations (9a–9c) are 1, triplicated $\lambda_{2}=-1/2$, and duplicated $\lambda_{1}$ and $\lambda_{3}$, under the limit s→0. It holds

$$K_{t}^{mm}\left(1122\right)=B_{13}+\left(B_{15}-\frac{C_{10}}{1-\lambda_{1}}\right){\left(\lambda_{1}\right)}^{t}+B_{16-17}{\left(\lambda_{2-3}^{t}\right)}^{T}+B_{18-20}t{\left(\lambda_{1-3}^{t}\right)}^{T}+B_{14}t^{2}{\left(\lambda_{2}\right)}^{t},$$ (A3.20a)

$$K_{t}^{mp}\left(1122\right)=C_{13}+\left(C_{15}-\frac{C_{10}}{1-\lambda_{1}}\right){\left(\lambda_{1}\right)}^{t}+C_{16-17}{\left(\lambda_{2-3}^{t}\right)}^{T}+C_{18-20}t{\left(\lambda_{1-3}^{t}\right)}^{T}+C_{14}t^{2}{\left(\lambda_{2}\right)}^{t},$$ (A3.20b)

$$K_{t}^{pp}\left(1122\right)=D_{13}+\left(D_{15}-\frac{C_{10}}{1-\lambda_{1}}\right){\left(\lambda_{1}\right)}^{t}+D_{16-17}{\left(\lambda_{2-3}^{t}\right)}^{T}+D_{18-20}t{\left(\lambda_{1-3}^{t}\right)}^{T}+D_{14}t^{2}{\left(\lambda_{2}\right)}^{t},$$ (A3.20c)

where $C_{10}$ is given by equation (A3.17), and the constant coefficients $B_{13-20}$, $C_{13-20}$, and $D_{13-20}$ are to be solved. Substituting $K_{t}^{mm}\left(1122\right)$ and $K_{t}^{mp}\left(1122\right)$ of equations (A3.20a, A3.20b) into the recurrence equation (9b), we obtain

$$\begin{array}{c} B_{13}=C_{13},\\ B_{14}=\left(2\lambda_{2}-1\right)C_{14},\\ B_{15-17}=\left(2\lambda_{1-3}-1\right)⊙C_{15-17}+2\lambda_{1-3}⊙C_{18-20}+\left(2C_{10},2\lambda_{2}C_{14},0\right),\\ B_{18-20}=\left(2\lambda_{1-3}-1\right)⊙C_{18-20}+\left(0,4\lambda_{2}C_{14},0\right). \end{array}$$ (A3.21)

Substituting $K_{t}^{mm}\left(1122\right)$ and $K_{t}^{pp}\left(1122\right)$ of equations (A3.20a, A3.20c) and $R_{t}^{m}$ of equation (A3.13) into the recurrence equation (9c), and substituting $B_{13-20}$ of equation (A3.21), we obtain

$$\begin{array}{c} D_{13}=C_{13}+s\left[\left(C_{8}+\frac{C_{10}}{1-\lambda_{1}}-C_{13}\right)\right],\\ D_{14}=\frac{\left(1-s\right)\left(2\lambda_{2}-1\right)}{\lambda_{2}}C_{14},\\ D_{15-17}=\left[\left(1-s\right)\left(2\lambda_{1-3}-1\right)⊘\lambda_{1-3}\right]⊙C_{15-17}+\left(1-s\right)C_{18-20}⊘\lambda_{1-3}\\ +\left(\frac{1-2s}{\lambda_{1}}C_{10},\frac{s}{\lambda_{2}}C_{9}-\frac{s}{\lambda_{2}}C_{11}-\frac{1-s}{\lambda_{2}}C_{14},\frac{s}{\lambda_{3}}C_{12}\right),\\ D_{18-20}=\left[\left(1-s\right)\left(2\lambda_{1-3}-1\right)⊘\lambda_{1-3}\right]⊙C_{18-20}+\left(0,\frac{s}{\lambda_{2}}C_{11}+2\frac{1-s}{\lambda_{2}}C_{14},0\right). \end{array}$$ (A3.22)

Substituting $K_{t}^{mp}\left(1122\right)$, $K_{t}^{mm}\left(1122\right)$ and $K_{t}^{pp}\left(1122\right)$ of equations (A3.20a–A3.20c) and $R_{t}^{m}$ of equation (A3.13) into the recurrence equation (9a), substituting $B_{13}-B_{20}$ of equation (A3.21), and substituting the exact $D_{13}-D_{20}$ of equation (A3.22), we obtain

$$C_{13}=C_{8}+\frac{C_{10}}{1-\lambda_{1}},C_{18}=-\left(1+\frac{8}{9}s\right)C_{10},C_{14}=C_{19}=C_{20}=0,$$ (A3.23)

where the constant coefficients $C_{14}$, $C_{19}$, and $C_{20}$ are approximated by keeping up to the zero order of *s*, while $C_{18}$ is approximated by keeping up to the first order of *s*. The theorem follows by substituting $B_{13-20}$, $C_{13-14}$, $C_{18-20}$, and $D_{13-20}$ of equations (A3.21–A3.23) into equations (A3.20a–A3.20c), where the constant coefficient $C_{15-17}$ of equation (A3.19) is determined by the initial conditions $K_{0}\left(1122\right)$. □

**Theorem A3.8.** *Denote by* $K_{t}\left(1232\right)={\left(K_{t}^{mm}\left(1232\right),K_{t}^{mp+}\left(1232\right),K_{t}^{pp}\left(1232\right)\right)}^{T}$ *the expected density of junction type* (1232) in a large population, and it holds

$$K_{t}\left(1232\right)=C_{4}\frac{\lambda_{1}^{t}-\lambda_{4}^{t}}{s}+P\Lambda \left(\lambda_{1-3}^{t}\right){\left(C_{21-23}\right)}^{T}+W_{t},$$ (A3.24)

*where* P is given by equation (A3.3), $C_{4-7}$ is given by equation (A3.11), *and*

$${\left(C_{21-23}\right)}^{T}=P^{-1}\left[K_{0}\left(1232\right)-W_{0}\right],$$ (A3.25)

$$\begin{array}{c} W_{t}=\left[\begin{array}{cccc} \frac{1}{3} & -\frac{2}{3s} & -\frac{7}{9} & -\frac{4}{9}\\ 0 & 0 & 0 & -\frac{4}{9}\\ -\frac{1}{3} & -\frac{4}{3s} & -\frac{20}{9} & \frac{32}{9} \end{array}\right]{\left[\left(\lambda_{1}^{t},\lambda_{2}^{t},\lambda_{3}^{t},\lambda_{7}^{t}\right)⊙C_{4-7}\right]}^{T}\\ +\left[\begin{array}{cc} -\frac{4}{3s} & -\frac{2}{9}\\ \frac{2}{3s} & \frac{4}{9}\\ \frac{8}{3s} & -\frac{8}{9} \end{array}\right]{\left[\left(t\lambda_{2}^{t},t\lambda_{3}^{t}\right)⊙C_{5-6}\right]}^{T}. \end{array}$$ (A3.26)

*Remark*. In equation (A3.24), $\left(\lambda_{1}^{t}-\lambda_{4}^{t}\right)/s\to 2t/3$ as s→0; the term $W_{t}$ of equation (A3.26) is independent of *s* since 1/s in the matrices is canceled with *s* of $C_{5}$ (see equation (A3.11)).

*Proof*. In a large population, the seven eigenvalues for the transition matrix of the linear recurrence equations (5a–5d) and equations (A1.41a–A1.41c) are $\lambda_{1}$, $\lambda_{4}$, $\lambda_{2}=\lambda_{5}=-1/2$, $\lambda_{3}=\lambda_{6}=1/4$, and $\lambda_{7}$. It holds

$$K_{t}^{mm}\left(1232\right)=B_{21-23}{\left(\lambda_{1-3}^{t}\right)}^{T}+B_{24}\left[{\left(\lambda_{4}\right)}^{t}-{\left(\lambda_{1}\right)}^{t}\right]+B_{25-26}t{\left(\lambda_{2-3}^{t}\right)}^{T}+B_{27}{\left(\lambda_{7}\right)}^{t},$$ (A3.27a)

$$K_{t}^{mp+}\left(1232\right)=C_{21-23}{\left(\lambda_{1-3}^{t}\right)}^{T}+C_{24}\left[{\left(\lambda_{4}\right)}^{t}-{\left(\lambda_{1}\right)}^{t}\right]+C_{25-26}t{\left(\lambda_{2-3}^{t}\right)}^{T}+C_{27}{\left(\lambda_{7}\right)}^{t},$$ (A3.27b)

$$K_{t}^{pp}\left(1232\right)=D_{21-23}{\left(\lambda_{1-3}^{t}\right)}^{T}+D_{24}\left[{\left(\lambda_{4}\right)}^{t}-{\left(\lambda_{1}\right)}^{t}\right]+D_{25-26}t{\left(\lambda_{2-3}^{t}\right)}^{T}+D_{27}{\left(\lambda_{7}\right)}^{t},$$ (A3.27c)

where the constant coefficients $B_{21-27}$, $C_{21-27}$, and $D_{21-27}$ are to be solved. Substituting $K_{t}^{mp+}\left(1232\right)$ and $K_{t}^{mm}\left(1232\right)$ of equations (A3.27a, A3.27b) and $\beta_{t}^{mmp}$ of equation (A1.16b) into the recurrence equation (A1.41b), we obtain

$$\begin{array}{c} B_{21}=\left(2\lambda_{1}-1\right)C_{21}+2\left(\lambda_{4}-\lambda_{1}\right)C_{24}-C_{4},\\ B_{22-23}=\left[2\lambda_{2-3}-1\right]⊙C_{22-23}+2\lambda_{2-3}⊙C_{25-26}-C_{5-6},\\ B_{24-27}=\left[2\lambda_{4-7}-1\right]⊙C_{24-27}+\left(-C_{4},0,0,-C_{7}\right). \end{array}$$ (A3.28)

Substituting $K_{t}^{mm}\left(1232\right)$ and $K_{t}^{pp}\left(1232\right)$ of equations (A3.27a, A3.27c) into the recurrence equation (A1.41c), we obtain

$$\begin{array}{c} D_{21}=\frac{\left(1-s\right)\left(2\lambda_{1}-1\right)}{\lambda_{1}}C_{21}-\frac{\left(1-s\right)\left(\lambda_{1}-\lambda_{4}\right)}{\lambda_{1}\lambda_{4}}C_{24}-\frac{1-s}{\lambda_{4}}C_{4},\\ D_{22-23}=\left[\left(1-s\right)\left(2\lambda_{2-3}-1\right)⊘\lambda_{2-3}\right]⊙C_{22-23}+\left(1-s\right)\left(C_{25-26}-C_{5-6}\right)⊘\lambda_{2-3},\\ D_{24-27}=\left[\left(1-s\right)\left(2\lambda_{4-7}-1\right)⊘\lambda_{4-7}\right]⊙C_{24-27}+\left(-\frac{1-s}{\lambda_{4}}C_{4},0,0,-\frac{1-s}{\lambda_{7}}C_{7}\right), \end{array}$$ (A3.29)

Substituting $K_{t}^{mm}\left(1232\right)$, $K_{t}^{mp+}\left(1232\right)$ and $K_{t}^{pp}\left(1232\right)$ of equations (A3.27a–A3.27c) and $\beta_{t}^{mmp}$ and $\beta_{t}^{mpp}$ of equations (A1.16b–A1.16c) into the recurrence equation (A1.41a), and substituting $B_{21-27}$ and $D_{21-27}$ of equations (A3.28, A3.29) we obtain

$$C_{24}=-\frac{B_{4}+2C_{4}}{3s},C_{25}=\frac{1}{9}\left(2B_{5}+C_{5}\right),C_{26}=-\frac{4}{9}\left(B_{6}-C_{6}\right),C_{27}=-\frac{4}{81}\left(4B_{7}+17C_{7}\right).$$ (A3.30)

According to equation (A3.10), we have

$$B_{4-7}=\left(1,\frac{3}{s},0,-2\right)⊙C_{4-7},$$ (A3.31)

and thus

$$C_{24-27}=\left(-\frac{1}{s},\frac{2}{3s},\frac{4}{9},-\frac{4}{9}\right)⊙C_{4-7},$$ (A3.32)

where $C_{4-7}$ is given by equation (A3.11). The constant coefficient $C_{21-23}$ is determined by the initial condition $K_{0}\left(1232\right)$, and it is given by equation (A3.25). The theorem follows by writing equations (A3.27a–A3.27c) in the matrix form while keeping only the leading order of *s*.

**Theorem A3.9.** *The expected density* $K_{t}^{mp-}\left(1232\right)$ in a large population is given by

$$K_{t}^{mp-}\left(1232\right)=\frac{1}{3}C_{4}{\left(\lambda_{4}\right)}^{t}+A_{28}{\left(\lambda_{5}\right)}^{t}+\frac{2}{3}C_{6}{\left(\lambda_{6}\right)}^{t}+\frac{4}{3}C_{7}{\left(\lambda_{7}\right)}^{t},$$ (A3.33)

*where* $C_{4}$, $C_{6}$, and $C_{7}$ are given by equation (A3.11), *and*

$$A_{28}=K_{0}^{mp-}\left(1232\right)-\frac{1}{3}C_{4}-\frac{2}{3}C_{6}-\frac{4}{3}C_{7}.$$ (A3.34)

*Proof*. In a large population, the eigenvalues of the transition matrix of the linear recurrence equations (5a–5d) and equation (A1.41d) are $\lambda_{4}$, duplicated $\lambda_{5}=-1/2$, $\lambda_{6}$ and $\lambda_{7}$. It holds

$$K_{t}^{mp-}\left(1232\right)=A_{24}{\left(\lambda_{4}\right)}^{t}+\left(A_{28}+A_{25}t\right){\left(\lambda_{5}\right)}^{t}+A_{26}{\left(\lambda_{6}\right)}^{t}+A_{27}{\left(\lambda_{7}\right)}^{t},$$ (A3.35)

where $A_{24-28}$ is to be solved. Substituting $K_{t}^{mp-}\left(1232\right)$ of equation (A3.35) and $\beta_{t}^{mmp}$ of equation (A3.9) into the recurrence equation (A1.41d), and we obtain

$$A_{24}=\frac{1}{3}C_{4},A_{25}=-C_{5},A_{26}=\frac{2}{3}C_{6},A_{27}=\frac{4}{3}C_{7},$$ (A3.36)

and $A_{28}$ is determined by the initial condition $K_{0}^{mp-}\left(1232\right)$ and it is given in equation (A3.34). The theorem follows by substituting equation (A3.36) into equation (A3.35), and approximating $A_{25}$ to zero since the leading term of $C_{5}$ is the first order of *s*, see equation (A3.11).

**Corollary A3.10.** *The overall expected unction density* $\rho_{t}^{mp}$ in a large population is given by

$$\begin{array}{c} \rho_{t}^{mp}=C_{8}+C_{10}\frac{1-\lambda_{1}^{t-1}}{1-\lambda_{1}}+\left[-C_{15}+\left(1+\frac{8}{9}s\right)C_{10}t\right]\lambda_{1}^{t}\\ +\left(-C_{16}-C_{9}-\frac{4}{3}C_{2}+\frac{2}{3}C_{2}t\right)\lambda_{2}^{t}+\left(-C_{17}-\frac{20}{9}C_{3}\right)\lambda_{3}^{t}, \end{array}$$ (A3.37)

*where* $C_{1-3}$, $C_{8-10}$, and $C_{15-17}$ are given by equation (A3.4), *equations (A3.15–A3.17)*, *and equation* (A3.19), *respectively*.

*Proof*. The corollary follows by substituting $R_{t}^{m}$ and $R_{t}^{p}$ of equations (A3.13, A3.14) and $K_{t}^{mp}\left(1122\right)$ of equation (A3.20b) into equation (3).
